# Clonal Heterogeneity Reflected by PI3K-AKT-mTOR Signaling in Human Acute Myeloid Leukemia Cells and Its Association with Adverse Prognosis

**DOI:** 10.3390/cancers10090332

**Published:** 2018-09-14

**Authors:** Ina Nepstad, Kimberley Joanne Hatfield, Tor Henrik Anderson Tvedt, Håkon Reikvam, Øystein Bruserud

**Affiliations:** 1 Section for Hematology, Department of Clinical Science, University of Bergen, 5021 Bergen, Norway; ina.nepstad@uib.no (I.N.); Kimberley.Hatfield@uib.no (K.J.H.); Hakon.Reikvam@uib.no (H.R.); 2 Departments of Immunology and Transfusion Medicine, Haukeland University Hospital, 5021 Bergen, Norway; 3 Section for Hematology, Department of Medicine, Haukeland University Hospital, 5021 Bergen, Norway; tor.henrik.anderson.tvedt@helse-bergen.no

**Keywords:** acute myeloid leukemia, PI3K, Akt, mTOR, phosphorylation, clonal heterogeneity

## Abstract

Clonal heterogeneity detected by karyotyping is a biomarker associated with adverse prognosis in acute myeloid leukemia (AML). Constitutive activation of the phosphatidylinositol-3-kinase-Akt-mechanistic target of rapamycin (PI3K-Akt-mTOR) pathway is present in AML cells, and this pathway integrates signaling from several upstream receptors/mediators. We suggest that this pathway reflects biologically important clonal heterogeneity. We investigated constitutive PI3K-Akt-mTOR pathway activation in primary human AML cells derived from 114 patients, together with 18 pathway mediators. The cohort included patients with normal karyotype or single karyotype abnormalities and with an expected heterogeneity of molecular genetic abnormalities. Clonal heterogeneity reflected as pathway mediator heterogeneity was detected for 49 patients. Global gene expression profiles of AML cell populations with and without clonal heterogeneity differed with regard to expression of ectopic olfactory receptors (a subset of G-protein coupled receptors) and proteins involved in G-protein coupled receptor signaling. Finally, the presence of clonal heterogeneity was associated with adverse prognosis for patients receiving intensive antileukemic treatment. The clonal heterogeneity as reflected in the activation status of selected mediators in the PI3K-Akt-mTOR pathway was associated with a different gene expression profile and had an independent prognostic impact. Biological heterogeneity reflected in the intracellular signaling status should be further investigated as a prognostic biomarker in human AML.

## 1. Introduction

Acute myeloid leukemia (AML) is a heterogeneous malignancy characterized by proliferating myeloblasts in the bone marrow [1,2,3]. Abnormal or constitutive signaling through intracellular pathways is often observed in the leukemic cells, including the phosphatidylinositol-3-kinase-Akt-mechanistic target of rapamycin (PI3K-Akt-mTOR) pathway that seems to be important both in normal and leukemic hematopoiesis [4,5,6]. Such abnormal signaling can be initiated by various mechanisms, e.g., various oncogenes or mutated receptor tyrosine kinases, cell adhesion molecules, G-protein-coupled receptors (GPCR) or other cytokine receptors.

Primary AML cell populations may include various subclones at the time of diagnosis, and relapse can occur due to regrowth of the originally dominating clone, a subclone detectable at the time of first diagnosis, or a new clone derived either from the original clone or from remaining preleukemic stem cells [7,8]. Detection of remaining leukemic cells with cytogenetic abnormalities at the time of allogeneic stem cell transplantation is associated with an adverse prognostic impact even when residual disease cannot be detected by flow cytometry, and morphological examination confirms that the patient is still in complete hematological remission [8]. One possible explanation for such a discrepancy may be the detection of a remaining minor subclone with a different immunophenotype compared with the dominating clone at the time of diagnosis [7]. The detection of subclones (i.e., clonal heterogeneity) based on karyotyping at the time of initial diagnosis is an independent adverse prognostic factor [9]. However, karyotyping for detection of clonal heterogeneity has several limitations as it is not a sensitive methodological approach and it cannot be used to detect heterogeneity for the majority of patients with normal karyotype or single cytogenetic abnormalities.

Clonal heterogeneity of AML cell populations can also be detected by flow cytometric analysis of constitutively activated intracellular signaling pathways, and this may therefore be an alternative methodological strategy [10] that can be used also for evaluation of the large group of patients with normal karyotype. Such analyses may also reflect a functional heterogeneity caused by driving mutations and thereby reflect characteristics that are important for leukemogenesis and/or chemosensitivity. In our present study, we therefore investigated how clonal heterogeneity is reflected in the activation status of the PI3K-Akt-mTOR pathway. This pathway was chosen because (i) it is usually activated in primary human AML cells; (ii) integrates signaling from a wide range of upstream mediators/receptors; (iii) shows crosstalk with and thereby also reflects the activation status of parallel intracellular pathways; and (iv) targets a wide range of downstream mediators that are important for essential cellular processes e.g., regulation of energy metabolism, gene transcription, protein synthesis, induction of apoptosis, and cellular communication [4,6,11]. In this context, we have therefore investigated samples from a group of 114 unselected patients to clarify whether analysis of constitutive PI3K-Akt-mTOR signaling can be used to detect clonal heterogeneity, whether such heterogeneity is a biomarker associated with any clinical or biological patient characteristics, and whether clonal heterogeneity has an independent prognostic impact.

## 2. Results

### 2.1. Clonal AML Cell Heterogeneity Reflected by PI3K-Akt-mTOR Signaling Is Seen for a Subset of Patients

In our flow cytometric analysis, we first identified the viable AML cells; this gating strategy is shown in Figure S1. The viable cell population was thereafter analyzed for expression levels of mediators and their phosphorylation. The viability of primary cells was analyzed both immediately after thawing and after the incubation steps by live dead gating. The viability did not differ significantly when comparing these two time points. The median frequency of dead cells after the incubation steps was 16.3% (range 0–48%). The viability of the AML cells did not differ when comparing AML cell samples with and without dual leukemic cell populations.

**Figure 1:**
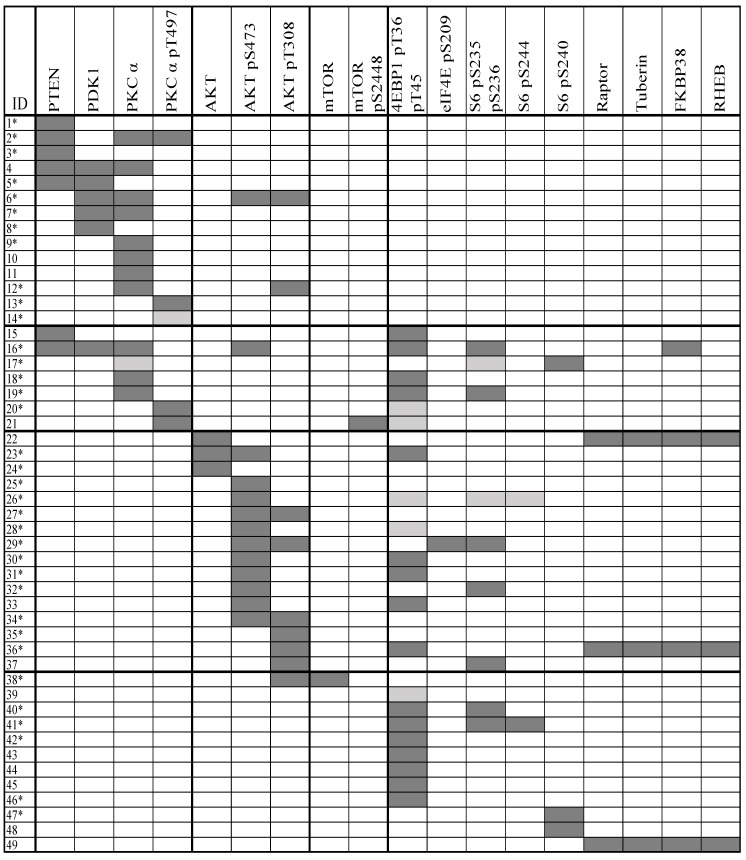
Clonal heterogeneity of primary human acute myeloid leukemia (AML) cell samples; an overview of the 49 patients showing dual populations when investigating activation of the phosphatidylinositol-3-kinase-Akt-mechanistic target of rapamycin (PI3K-Akt-mTOR) pathway. The cells were incubated in medium alone (all patients), with insulin alone and with insulin and a pathway inhibitor (rapamycin, GDC-0941; only an unselected subset of patients). Dark grey means that dual populations were detected in all cultures with and without treatment, and light grey indicates detection only for some cultures. Patient samples analyzed for inhibition by mTOR inhibitor rapamycin and PI3K inhibitor GDC-0941 are indicated by an asterisk (*).

We investigated the 18 mediators from the PI3K-Akt-mTOR pathway in leukemic cell samples from 114 unselected AML patients. Dual populations were detected in samples from 49 of patients and these overall results are summarized in Figure 1. The flow cytometric evidence for clonal heterogeneity is presented more in detail in Figure S1, and it can be seen that a minor population was clearly separated from the main AML cell population for all the 49 patients.

For each of the 49 patients, clonal heterogeneity was detected only for some of the 18 mediators. Nineteen of the patients showed dual population for only one of any of the 18 mediators, 16 patients showed dual populations for two mediators, and dual populations for three mediators was found for seven patients. Thus, for the majority of these patients, dual AML cell populations were detected only by one or a few of the tested mediators, and dual populations for four to seven pathway mediators were observed only for seven patients.

Dual populations for mediators upstream to Akt were detected for 20 patients, whereas dual populations for Akt and mTOR were only detected for four of these 20 patients. Dual populations for mediators downstream to mTOR were detected for 10 patients. Furthermore, clonal heterogeneity reflected in constitutive Akt activation but not reflected by mediators upstream to Akt was found in 17 patients. This heterogeneity reflects differences in activation alone because the total protein levels of Akt did not differ between cell subsets. Finally, it was uncommon for mTOR total protein level or phosphorylation/activation to reflect clonal heterogeneity.

Taken together, the observations described above show that 49 out of 114 patients with clonal heterogeneity differ with regard to how the heterogeneity is reflected in the PI3K-Akt-mTOR expression/phosphorylation profile.

The patient subsets with and without clonal heterogeneity were also compared with regard to their molecular genetics (i.e., analysis of 54 different mutations), morphological as well as molecular signs of differentiation and the overall PI3K-Akt-mTOR activation profile; though the two patient subsets did not show any significant differences with regard to these biological characteristics (see Table S1).

### 2.2. Primary AML Cells Derived from Patients with and without dual PI3K-Akt-mTOR Cell Populations Differ in Their Global Gene Expression Profiles

**Figure 2:**
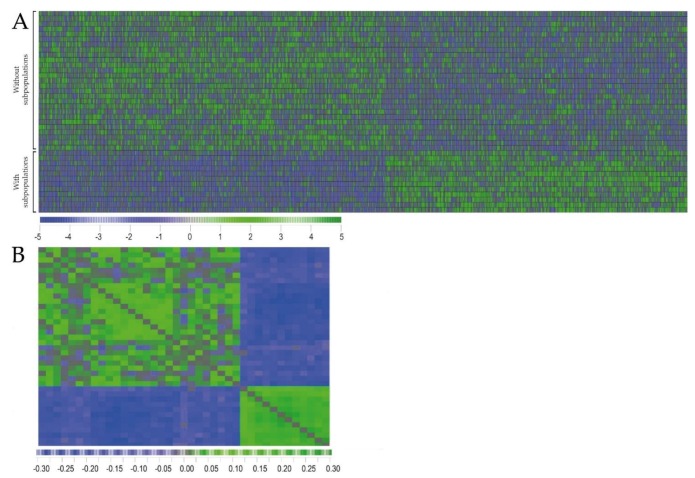
Comparison of global gene expression profiles and gene ontology for primary human AML cells with and without clonal heterogeneity based on the analysis of PI3K-Akt-mTOR activation. Global gene expression profiles (GEP) were available for 39 unselected AML patients included in the study, 12 samples were derived from patients showing dual cell populations and 27 leukemic cell populations did not show dual cell populations. (**A**) A feature subset selection (FSS) was performed to identify the most discriminative genes between the two groups, and 1209 genes were identified (*p*-value <0.05). These genes were used in a hierarchical clustering model (Pearson’s correlation distance measure with complete linkage) demonstrating a highly discriminative expression pattern for the groups with and without subclones. (**B**) A correlation visualization with distance matrix displays the pairwise correlation between the 39 patients. Blue and green colors highlight the negative and positive correlation between samples. The genes found differently expressed were thereafter used to identify gene ontology terms (using the David Database for Gene Ontology) that were overrepresented among the genes differently expressed. A more detailed version is shown in Figure S3.

We compared the global gene expression profiles for 12 AML samples with and 27 samples without dual AML cell populations. A feature subset selection (FSS) analysis was performed for identification of the most discriminative genes between the two groups, and 1209 genes were then identified (i.e., *p*-value <0.05, see the complete gene list is included in Table S5 and Table S6). This criterion was chosen because AML is a highly heterogeneous disease with regard to differentiation, karyotype and molecular genetics [1,2,3]; and this can be seen from the characteristics of our present patient cohort (Table 1 and Table 2 below, Table S1). For this reason, a relatively large number of differentially expressed genes will probably be needed to distinguish between patient subsets. The identified genes were used in a hierarchical clustering model (Pearson’s correlation distance measure with complete linkage; and this analysis identified two main patient subsets that corresponded to the patients with and without subpopulations. Furthermore, a correlation visualization with distance matrix displays the pairwise correlation between the 39 patients and the classification of patients into two main subsets corresponding to the subsets with and without subclones, respectively (Figure 2).

The differentially expressed genes were thereafter used to identify gene ontology terms that were overrepresented among the differentially expressed genes. We then selected Gene Ontology (GO) terms based on the criteria (i) False Discovery Rate (FDR) ad modum Benjamini <0.05 and (ii) *p*-values <0.05. Only two GO-terms were identified in each of the two analyses based on Biological processes or Molecular function, respectively. Based on Molecular function, the two groups G-protein coupled receptor signaling pathway (*p* = 0.0000078; 43 genes included) and Detection of chemical stimulus involved in sensory perception of smell (*p* = 0.000015; 26 genes included) were identified. Based on analysis of Biological processes we identified the two groups G-protein coupled receptor activity (*p* = 0.00002; 36 genes included) and Olfactory receptor activity (*p* = 0.000024; 26 genes included). The complete gene lists for each of these four GO terms are presented in Tables S2 and S3. None of the two patient subsets showed generally higher expression for all differentially expressed genes, but the two subsets differ in their pattern of high/low expressed genes.

### 2.3. Detection of AML Subclones Based upon PI3K-Akt-mTOR Signaling Is Associated with Decreased Patient Survival

**Figure 3:**
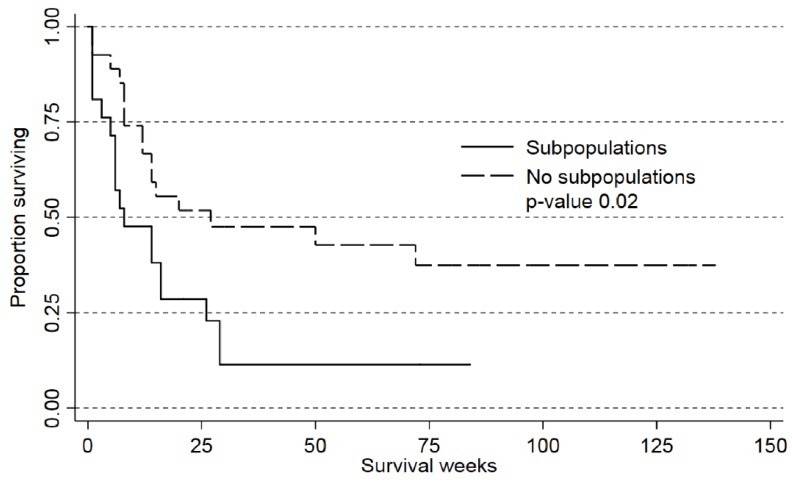
Overall survival of patients with (17 patients) and without (27 patients) clonal heterogeneity when investigating PI3K-Akt-mTOR pathway activation; an analysis of 44 patients who completed their intensive induction treatment followed by at least two consolidation cycles, autologous or allogeneic stem cell transplantation after their first diagnosis of AML.

In a recent study, karyotyping could detect clonal heterogeneity for approximately 15% of patients, and this heterogeneity was then associated with an adverse prognosis in patients receiving intensive AML therapy [9]. We therefore investigated whether detection of subclones determined by analysis of constitutive PI3K-Akt-mTOR activation had a similar prognostic association. In our present study, we investigated a large group of unselected patients that included several elderly and unfit patients who could not receive intensive treatment [12]. However, 44 of our patients completed their planned intensive induction treatment followed by at least two consolidation cycles, autologous or allogeneic stem cell transplantation after their first diagnosis of AML. We compared the survival of 17 patients with and 27 patients without subpopulations; the overall survival was significantly higher for patients without subpopulations (Figure 3; *p* = 0.027). This association between prognosis and heterogeneity was significant both in crude (Table 1, Cox Proportional Hazard Model, *p* = 0.03) and adjusted analyses (*p* = 0.04). To summarize, the previous study [9] showed that detection of clonal heterogeneity in patients with abnormal karyotype had an adverse prognostic impact. In our present study we used an alternative methodological strategy for detection of subclones independent of the karyotype (i.e., also including patients with normal karyotype), and we could detect an association between clonal heterogeneity and adverse prognosis.

We also did an adjusted analysis of the prognostic impact of age, clonal heterogeneity, cytogenetics, *Flt3/NPM1* status (Table 1). After correcting for these adverse factors, clonal heterogeneity still had a significant association with survival.

## 3. Discussion

The PI3K-Akt-mTOR pathway shows constitutive activation in human AML and is therefore regarded as a possible therapeutic target, but despite this, the results from initial clinical studies suggest that pathway inhibitors have only modest antileukemic activity [13]. Possible explanations for this could be that patients are heterogeneous with regard to their susceptibility [14] due to differences in the crosstalk with other pathway [15], or there is clonal heterogeneity with variation in constitutive pathway activation between leukemic subclones for individual patients [7]. In the present study, we used flow cytometric analysis of PI3K-Akt-mTOR activation to detect clonal heterogeneity. We investigated a large group of samples derived from unselected AML patients (i.e., the large majority of the patients had normal karyotype or only a single cytogenetic abnormality), and clonal heterogeneity was detected for the majority of these patient samples. However, for each of these patients the clonal heterogeneity was reflected in the basal expression of only one or a few of the 18 investigated pathway mediators, i.e., this heterogeneity was not associated with a difference in activation status throughout the pathway. A possible explanation for this limited pathway heterogeneity could be that the activation status of each mediator not only reflects the downstream signaling from receptor ligation, but also the crosstalk between specific mediators of the PI3K-Akt-mTOR pathway and neighboring intracellular pathways.

Most of our patients were elderly or unfit patients that could not receive intensive antileukemic treatment. Our patients are thus representative with regard to AML cell biology, but they are heterogeneous with regard to antileukemic treatment and the elderly/unfit patients usually received only disease-stabilizing or supportive treatment [12].

Aberrant expression of lymphoid markers is relatively common in AML, and according to the World Health Organization (WHO) classification an uncommon subset of acute leukemia patients also shows a mixed phenotype with both myeloid and lymphoid leukemic cell subpopulation [1]. However, among our heterogeneous AML cell populations neither patients with mixed leukemic phenotype nor aberrant expression of lymphoid markers (CD2, CD3, CD4, CD8, CD19, CD20) were detected. Furthermore, the presence of clonal heterogeneity in the PI3K-Akt-mTOR pathway showed no association with cytogenetic abnormalities, mutational status, morphological or molecular signs of differentiation. Thus, our identification of two patient subsets with and without clonal heterogeneity based on pathway activation seems independent of the conventional subclassification of AML patients.

Clonal heterogeneity can be detected by various methodological strategies [7,9,10], including karyotyping which identified 15% of patients with clonal heterogeneity [9]. We observed clonal heterogeneity for 49 out of 114 patients (42%) using our flow cytometric approach and this higher frequency is most likely due to an additional molecular heterogeneity not reflected by karyotyping. 

We used flow cytometry to evaluate constitutive PI3K-Akt-mTOR activation and the criteria for detection of two AML cell subpopulations were (i) two distinct and clearly separated cell subsets for one or more of the 18 mediators; and (ii) the smallest subpopulation being at least 20% of the total viable cell population. Considering the limited number of metaphases analyzed by karyotyping, the study by Bochtler et al. [9] suggests that the clonal heterogeneity has to reach a certain (i.e., detectable) level to have a prognostic impact. By using 20% as our cutoff it was possible to identify distinct cell populations, and this cutoff has also been used to define positivity for differentiation markers by flow cytometry [16].

PI3K-Akt-mTOR is a part of a complex signaling network involving several single mediators and showing crosstalk with other pathways [13]. We selected 18 flow cytometric parameters that reflect the status of the main track of the pathway, including absolute levels and phosphorylation status of important upstream mediators, the key mediators Akt and mTOR and mediators downstream to mTOR (Table S1). It can be argued that for example phosphorylated PTEN should also be included, even though this mediator may be less important in AML than in many other malignancies at least with regard to PTEN mutations; PTEN seems to be mutated in less than 1% of AML patients. However, PDK1 is located between PI3K and Akt in the main pathway track, and the activity of PTEN will be reflected in the immediately downstream PDK1 phosphorylation [4].

The viability of the cryopreserved cells after thawing was determined for all patients and despite a variation between patients, all samples showed more than 50% viable cells. One would expect less than 100% viability for most patients when testing cryopreserved cells [17] and the viability did not differ between patients with and without detectable subclones. Thus, the detection of subclones is not associated with altered susceptibility of the AML cells to stress-induced or spontaneous in vitro apoptosis.

The AML cell population from a single patient may consist of various subclones [7,9] that can be detected by single cell analyses (i.e., flow cytometry) of constitutive pathway activation [10]. We never observed more than two subsets (dual populations) in a patient sample, independently of which mediator was analyzed. However, our methodological approach does not allow an accurate estimation of the total number of subpopulations when dual populations were detected for two or more pathway mediators because the same two subpopulations may be detected when analyzing various pathway mediators, or different subpopulations identified by different mediators. For this reason, we could classify our patients as either showing or not showing clonal heterogeneity, but we could not estimate the number of subclones in this assay.

We compared the global gene expression profiles for AML cell samples with and without clonal heterogeneity; gene expression data were then available only for an unselected subset of our patients. We first investigated whether gene expression profiling could be used to identify patients with and without detectable clonal heterogeneity in the flow cytometric analysis. AML is a very heterogeneous disease [12], and as would be expected the patient heterogeneity is also illustrated by the clinical and biological characteristics (Table 1 and Table 2, Table S2) of the patients included in our present study. We performed a clustering analysis (Figure 2) based on the differentially expressed genes and identified two patient subsets corresponding to the patient samples with and without dual subpopulations. Thus, despite the extensive heterogeneity of the AML disease, the patient subsets that are showing dual populations can be identified by analysis of gene expression profiles.

To further investigate the biological differences between patients with and without clonal heterogeneity, we performed a GO term analysis, and we then identified the terms with FDR <0.05 and statistical significance with *p* <0.05. This analysis was based on a correction for multiple hypothesis testing and ontologies including few genes were left out. We identified only two GO-terms both when the analysis was based on Biological processes (G-protein receptor signaling, Detection of stimulus smell) and on Molecular function (G-protein receptor activity, Olfactory receptor activity).

Our present studies showed that patients with and without clonal heterogeneity differed with regard to the expression of genes encoding olfactory receptor components and proteins involved in downstream signaling from G-protein coupled receptors (GPCRs) signaling. The olfactory receptors are a subset of the GPCRs [18,19,20]. Olfactory receptors are expressed in many tissues and by many different normal cells outside the olfactory system. This includes several normal leukocytes (e.g., monocytes, neutrophil granulocytes, erythrocytes, T and B cells, NK cells) [21] as well as adipose tissue, heart, skeletal muscles, kidney, prostate, gastrointestinal tract, liver, lung, several endocrine organs, ovary and testes [22]. It can also be expressed by various malignant cells [23,24,25,26,27,28], including human AML cells [29,30]. Thus, both normal and malignant myeloid cells are among the cells that show ectopic expression of olfactory receptors, but to the best of our knowledge, our present study is the first to describe an association between olfactory receptor expression and prognosis in a hematological malignancy. Furthermore, within these tissues certain olfactory receptors seem to have a more limited expression, whereas other receptors have a more widespread expression [22]. Limited data are available for the functional roles of ectopic olfactory receptors, but the overall data suggest that they can be involved in modulation and regulation of important cellular processes like cell survival/apoptosis induction, cell-cell recognition, proliferation and migration [22,28]. For cancer cells, these receptors seem to influence migration and development of metastases from solid tumors [31,32,33,34]. Our present study is one of the first to describe the broad olfactory receptor expression by primary human AML cells, and to the best of our knowledge, it is the first to suggest an association between chemosensitivity/survival in a hematological malignancy.

Ectopically expressed olfactory receptors influence signaling through several signal transduction pathways, including the PI3K-Akt-mTOR pathway as well as NFκB, MEK-ERK1/2 and p42/44, and they seem to regulate calcium metabolism [22]. Several of these pathways are also important in AML [35]. The bone marrow ligands of these receptors are not known, but one possibility is binding of various metabolites [36]. Several metabolites and metabolic intermediates are ligands for olfactory receptors, including lactate, short- and medium-chain fatty acids, ketones and steroids [22,28,37]. Other metabolic intermediates share structural similarities with known ligands [22,28]. Our hypothesis is that the ectopic olfactory receptors function as metabolic sensors and the metabolic/metabolite profile of the bone marrow microenvironment thereby becomes important for leukemogenesis and/or AML cell chemosensitivity. The local metabolic profile will probably also be influenced by the systemic metabolic profile, and this may explain why differences in the systemic (i.e., serum) metabolite profile has a prognostic impact in human AML [38]. Finally, these receptors can also be expressed by various stem cells [39], but their expression at the protein level has not been characterized, and it is not known whether they are expressed by leukemic or normal hematopoietic stem cells either. However, the observation that ectopic olfactory receptors are expressed at all stages of erythroid cell development [28,40] suggests that they have a more widespread expression at least in normal hematopoietic cells.

Clonal heterogeneity detected by cytogenetic analysis has an adverse prognostic impact [9]. We used a methodological approach that enabled us to investigate all patients with respect to clonal heterogeneity, including the large group of patients with normal karyotype and the same single abnormality in all investigated AML cells. Our present analysis also showed an association between clonal heterogeneity and adverse prognosis, and clonal heterogeneity was an independent prognostic parameter in our present study of a patient cohort mainly including patients with normal karyotype or favorable genetic abnormalities and only a small subset of patients having a complex karyotype.

Karyotyping is not suitable for rapid detection of clonal heterogeneity in routine clinical practice and this methodological strategy cannot be used for the majority of patients, e.g., patients with single abnormalities or normal karyotype, whereas our present strategy based on flow cytometry can detect clonal heterogeneity within a few hours. Our present study identifies detection of heterogeneity in PI3K-Akt-mTOR activation as a possible biomarker with prognostic impact in human AML. However, the activation status of this pathway may not only be used as a biomarker; the pathway is involved in several important cellular functions and therefore it may be a possible therapeutic target in human AML.

## 4. Materials and Methods

### 4.1. AML Patients

The study was approved by the Regional Ethics Committee (REK) (REK III 060.02, 10 June 2002; REK Vest 215.03, 12 March 2004; REK III 231.06, 15 March 2007; REK Vest 2013/634, 19 March 2013; REK Vest 2015/1410, 19 June 2015), The Norwegian Data Protection Authority 02/1118-5, 22 October 2002, and The Norwegian Ministry of Health 03/05340 HRA/ASD, 16 February 2004. All AML cell samples were collected after written informed consent. The clinical and biological characteristics of the 114 unselected patients included in the study are summarized in Table 2 (49 females and 65 males; median age 67 years with range 18–87 years). All patients had a high number and/or percentage of peripheral blood blasts; leukemic peripheral blood mononuclear cells could therefore be isolated by density gradient separation alone (Lymphoprep, Axis-Shield, Oslo, Norway) and generally contained at least 95% leukemic blasts. The contaminating cells were small lymphocytes. These enriched AML cells were stored in liquid nitrogen until used in the experiments [41].

### 4.2. Flow-Cytometric Analysis of PI3K-Akt-mTOR Activation

Pathway-associated clonal heterogeneity was defined as flow-cytometric detection of at least one extra cell population including at least 20% of the leukemic cells and being detection by the analysis of at least one of the PI3K-Akt-mTOR mediators.

Flow cytometry was used to examine the constitutive expression of 18 mediators in the PI3K-Akt-mTOR pathway/network in the AML cells. Cryopreserved and thawed primary leukemic cells were incubated for 20 minutes in RPMI-1640 (Sigma-Aldrich, St. Louis, MO, USA) before being directly fixed in 1.5% paraformaldehyde (PFA) and permeabilized with 100% ice-cold methanol. The cells were thereafter rehydrated by adding 2 mL phosphate-buffered saline (PBS), gently re-suspended and then centrifuged. The cell pellet was washed twice with 2 mL PBS and resuspended in 150 μL PBS supplemented with 0.1% bovine serum albumin (BSA) (Sigma, St. Louis, MO, USA). Washed cells were blocked with immunoglobulin (Octagam; Octapharma, Jessheim, Norway) and 1% BSA, and thereafter split evenly into nineteen new tubes (1 × 10^5^ cells per sample) before staining. All staining panels included the same live/dead discriminator, either FITC or Alexa Fluor^®^ 647 Mouse anti-Cleaved PARP (Asp214); a blank sample was also included. Three directly conjugated dyes were used (Table S4): (i) Alexa Fluor^®^ 647 was used for PTEN, PDPK1 (pS241), PKCα, PKCα (pT497), Akt (pS473), 4EBP1 (pT36/pT45), elF4E (pS209), S6 (pS244), and mTOR; (ii) phycoerythrin (PE) for Akt total, Akt (pT308), mTOR (pS2448), and S6 (pS240); and (iii) V450 for S6 (pS235/pS236). Antibodies were purchased from BD Pharmingen (Franklin Lakes, NJ, USA) except for anti-mTOR that was purchased from Cell Signal Technology (Danvers, MA, USA). Four of the antibodies were unconjugated (anti Raptor, Tuberin, FKBP38, and RHEB; all from Abcam; Cambridge, UK) and required secondary antibody-conjugated Alexa Fluor^®^ 647 (Franklin Lakes, NJ, USA). Together all these mediators represent main steps in the PI3K-Akt-mTOR pathway and they were selected to provide an extended phosphorylation profile of this pathway.

Viable leukemic cells were identified and analyzed in flow cytometric analysis based on live/dead staining, doublet discrimination, CD45 staining and forward/side scatter. Dead cells, doublets and the minor contaminating lymphocyte populations were excluded from further analyses. Our flow cytometric analyses were thus based on the analysis of viable leukemic cells, and the gating strategy used to identify the viable cell population is shown in Figure S2.

AML cells were also incubated with human insulin 10 µg/mL (Sigma-Aldrich, St. Louis, MO, USA); the non-specific mTOR inhibitor rapamycin (LC Laboratories, Woburn, MA, USA) and the PI3K class I specific inhibitor GDC-0941 (Axon Medchem BV, Groningen, Netherlands) were added at a final concentration of 100 nM. Cells were incubated with these agents for 15 minutes before fixation/staining and flow cytometric analysis as described above.

### 4.3. Analysis of Global Gene Expression Profiles and Mutation Analyses

Our methods for RNA preparation, labelling and microarray hybridization have been described in detail previously [42]. All microarray experiments were performed using the Illumina iScan Reader, which is based upon fluorescence detection of biotin-labelled cRNA that was hybridized to the HumanHT-12 V4 Expression BeadChip according to the manufacturer’s instructions. The HumanHT-12 V4 BeadChip targets 47 231 probes that are mainly derived from genes in the NCBI RefSeq database (Release 38). Preprocessing, normalization and annotations of the microarray data has also been described in detail in this previous publication; the data from the array scanning were investigated in GenomeStudio and J-Express 2012 for quality control measures.

Submicroscopic mutation profiling of 54 genes frequently mutated in myeloid leukemias was done using the Illuminas TruSight Myeloid Gene Panel and sequenced using the MiSeq system and reagent kit v3 (all from Illumina, San Diego, CA, USA) as described in detail previously [42]. The methods for fragment analysis of *Flt3* exon 14–15 and *NPM1* exon 12 and analysis of *CEBPA* mutations have also been described previously [42].

### 4.4. Data Collection, Bioinformatical and Statistical Analyses

Flow cytometry analysis was acquired on a BD FACSVerse 8-color flow cytometer (BD Biosciences; Franklin Lakes, NJ, USA) and data analysis performed using FlowJo 10.0.7 software (Tree Star, Inc., Ashland, OR, USA). The J-Express software (J-Express 2012, MolMine AS, Bergen, Norway) was used for bioinformatical analyses. For unsupervised hierarchical clustering analysis, all values were calculated using fold change on the Inverse hyperbolic sine (Arcsinh) scale and with median values for each target group as control. Complete linkage (euclidean distance) and Pearson correlation were used as linkage method and distance measurement, respectively. Statistical analyses were performed using the IBM Statistical Package for the Social Sciences (SPSS) version 23 (IBM Corporation, Armonk, NY, USA). The Mann-Whitney *U*-test was used to compare different groups; the Chi square/Fischer’s exact test for analysis of categorized data and the Kendall’s tau-b correlation test for correlation analyses. The Cox Proportional Hazard Model was used for analysis of survival data. *p*-values <0.05 were regarded as statistically significant. The gene ontology enrichment analysis of differentially expressed genes was performed by using online bioinformatics tools of DAVID Bioinformatics Resources 6.8, Laboratory of Human Retrovirology and Immunoinformatics (LHRI) [43,44].

## 5. Conclusions

To conclude, clonal heterogeneity in human AML cell samples is reflected in the activation of mediator in the PI3K-Akt-mTOR pathway, and this heterogeneity had an independent prognostic impact in our patient cohort. Our present study suggests that that clonal heterogeneity as reflected in intracellular signaling pathways should be further investigated as a possible adverse prognostic biomarker in future clinical studies.

## Figures and Tables

**Table 1:** A comparison of overall survival for patients with and without dual populations based on the analysis of PI3K-Akt-mTOR activation. The table presents the results from univariate and adjusted or multivariate analyses (Cox Proportional Hazard Model). Significant *p*-values in the adjusted analysis are marked in bold.

Covariate	Crude Analysis			Adjusted Analysis		
	*p*-Value	HR	95%-CI	*p*-Value	HR	95%-CI
Age (per decade)	<0.01	1.64	1.24–2.17	<0.01	1.69	1.22–2.36
Subpopulation versus no subpopulation	0.03	2.15	1,01–4,26	0.04	2.28	1.03–5.04
Adverse cytogenetics	0.519	0.759	0.33–1.75	0.11	0.33	0.11–1.04
NPM1-wt and Flt3-wt	NA	1 (reference)		NA	1 (reference)	
NPM1-mutated and Flt3-wt	0.92	1.62	0.58–4.56	0.693	1.24	0.42–3.75
NPM1-wt and FLT3-mutated	0.21	1.86	0.71–4.92	0.03	3.88	1.18–12.71
NPM1-mutated and FLT3-mutated	0.05	2.33	1.00–5.34	0.48	1.44	0.57–3.61

Abbreviations: CI, confidence interval; HR, hazard ratio. NPM1-wt: Nucleophosmin 1-wild type; Flt3-wt: fms-like tyrosine kinase 3-wild type.

**Table 2:** The biological and clinical characteristics of the 114 AML patients included in the study.

Patient characteristics			
Age		Secondary AML	
Median (years)	67	chemo	5
Range (years)	18–87	CML	2
		CML-RELAPSE	1
Gender		CMML	4
Females	49	*de novo*	81
Males	65	LiFraum, chemo	1
		MDS	8
		MDS, AML relapse	1
		MDS, CHEMO	1
		Myelofibrosis	3
		Polycytemia vera	1
		Relapse	5
Total	114	Relapse, chemo	1
**Cell Morphology**			
**FAB Classification**		**CD34 Receptor**	
M0	8	Negative (<20%)	30
M1	28	Positive (>20%)	78
M2	22	n.d.	6
M4	27		
M5	21		
M7	1		
n.d.	7		
**Cell Genetics**			
**Cytogenetics**		**Mutations**	
Adverse	20	*NPM1* mutationsMutated	35
Favorable	11	Wild-type	62
Intermediate	11	n.d.	17
Normal	60		
n.d.	12	*Flt3* mutations	
		ITD	41
		Wild-type	55
		n.d.	18

The European Leukemia Net classification was used; n.d.: not determined; CML: Chronic myeloid leukemia; CMML:Chronic myelomonocytic leukemia; FAB: French-American-British.

## Supplementary Materials

The following are available online at http://www.mdpi.com/2072-6694/10/9/332/s1. Figure S1: Detection of clonal heterogeneity for 49 AML patients; Figure S2. Cell preparation and gating strategy; Figure S3. A correlation visualization with distance matrix; Table S1. A comparison of patients with and without dual AML cell populations; studies of molecular genetics, differentiation and pathway activation profiles; Table S2. The GO-term showing significant differences when comparing AML cell populations for patients with and without clonal heterogeneity; the comparison being based on analysis of Molecular function; Table S3. The GO-term showing significant differences when comparing AML cell populations for patients with and without clonal heterogeneity; Table S4. Monoclonal antibodies used in the flow-cytometric studies; Table S5. The complete gene list from patients with dual AML cell populations; Table S6. The complete gene list from patients without dual AML cell populations.
