# Supplementary material for: Clonal Heterogeneity Reflected by PI3K-AKT-mTOR Signaling in Human Acute Myeloid Leukemia Cells and Its Association with Adverse Prognosis
Source: Cancers (Basel). 2018 Sep 14;10(9):332. doi: 10.3390/cancers10090332 (PMC6162751; doi:10.3390/cancers10090332)
Supplement: Supplementary file 1 [file cancers-10-00332-s001.pdf]

Supplementary Materials: Clonal Heterogeneity Reflected by PI3K-AKT-mTOR Signaling in Human Acute Myeloid Leukemia Cells and its Association with Adverse Prognosis

Ina Nepstad, Kimberley Joanne Hatfield, Tor Henrik Anderson Tvedt, Håkon Reikvam and Øystein Bruserud

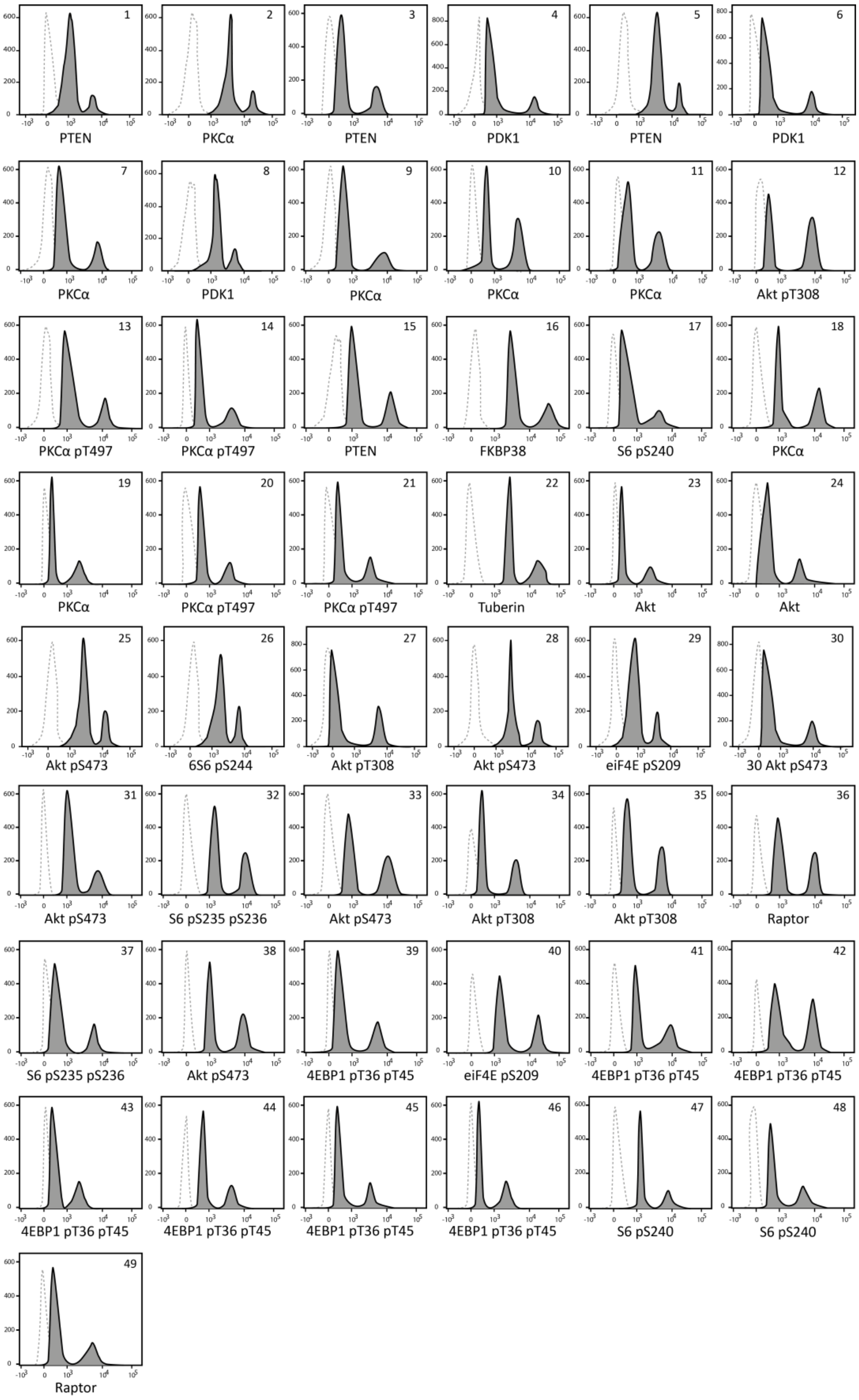

Figure S1. Detection of clonal heterogeneity for 49 acute myeloid leukemia (AML) patients; the results from representative flow cytometric analyses of phosphatidylinositol-3-kinase-Akt-mechanistic target of rapamycin (PI3K-Akt-mTOR) activation. For each patient clonal heterogeneity was detected by analysis

of at least one mediator in the PI3K-Akt-mTOR pathway. Patient ID is shown in the upper right corner of each histogram. The figure documents the detection of dual populations for all patients, showing the results from one representative flow cytometric analysis for each of these 49 patients. The Y-axis represents the amount of cells, and the X-axis represents the fluorescence intensity. The stippled line shows the negative/unstained controls.

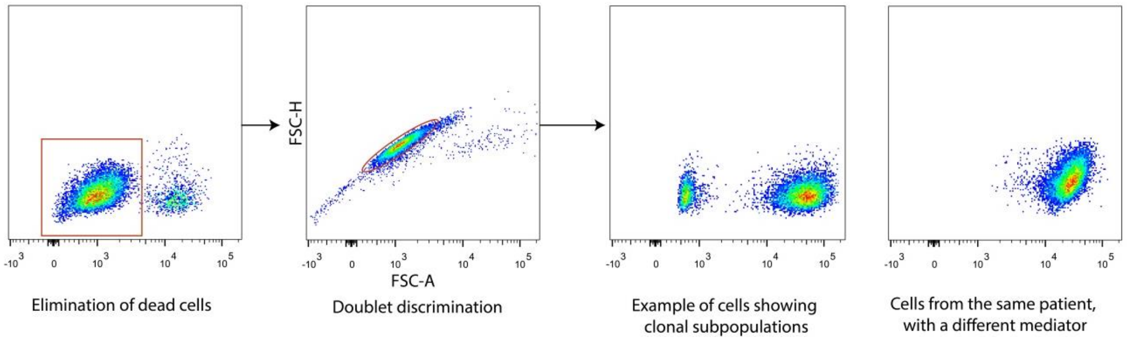

Figure S2. Cell preparation and gating strategy. Flow cytometry was used for examination of the constitutive expression of the mediators in the PI3K-Akt-mTOR pathway/network in primary AML cells. Cryopreserved cells were thawed and washed before suspension cultures were prepared as described in Materials and methods. Briefly, cryopreserved and thawed primary leukemic cells were incubated for 20 minutes in RPMI-1640 (Sigma-Aldrich) before being directly fixed in 1.5% paraformaldehyde (PFA) and permeabilized with 100% ice-cold methanol. The cells were thereafter rehydrated by adding 2 mL phosphate buffered saline (PBS), gently re-suspended and then centrifuged. The cell pellet was washed twice with 2 mL PBS and resuspended in 150 µL PBS supplemented with 0.1% bovine serum albumin (BSA) (Sigma, St. Louis, Missouri). Washed cells were blocked with immunoglobulin (Octagam; Octapharma, Jessheim, Norway) and 1% bovine serum albumin (BSA), and thereafter split evenly into nineteen new tubes (1 x 10^5 cells per sample) before staining. The first step in the gating was to distinguishing populations of cells based on their forward and side scatter properties. Doublet discrimination was performed based on both forward and side scatter, and elimination of dead cells was performed based on the live/dead discriminator, either FITC or Alexa Fluor® 647 Mouse anti-Cleaved PARP (Asp214). The identified viable cells were further analyzed based on each of the mediators/antibodies used in the study. The colors refer to the density of the cells relative to one another, where blue and green correspond to areas of lower cell density, yellow is mid-range, and red and orange are areas of high cell density.

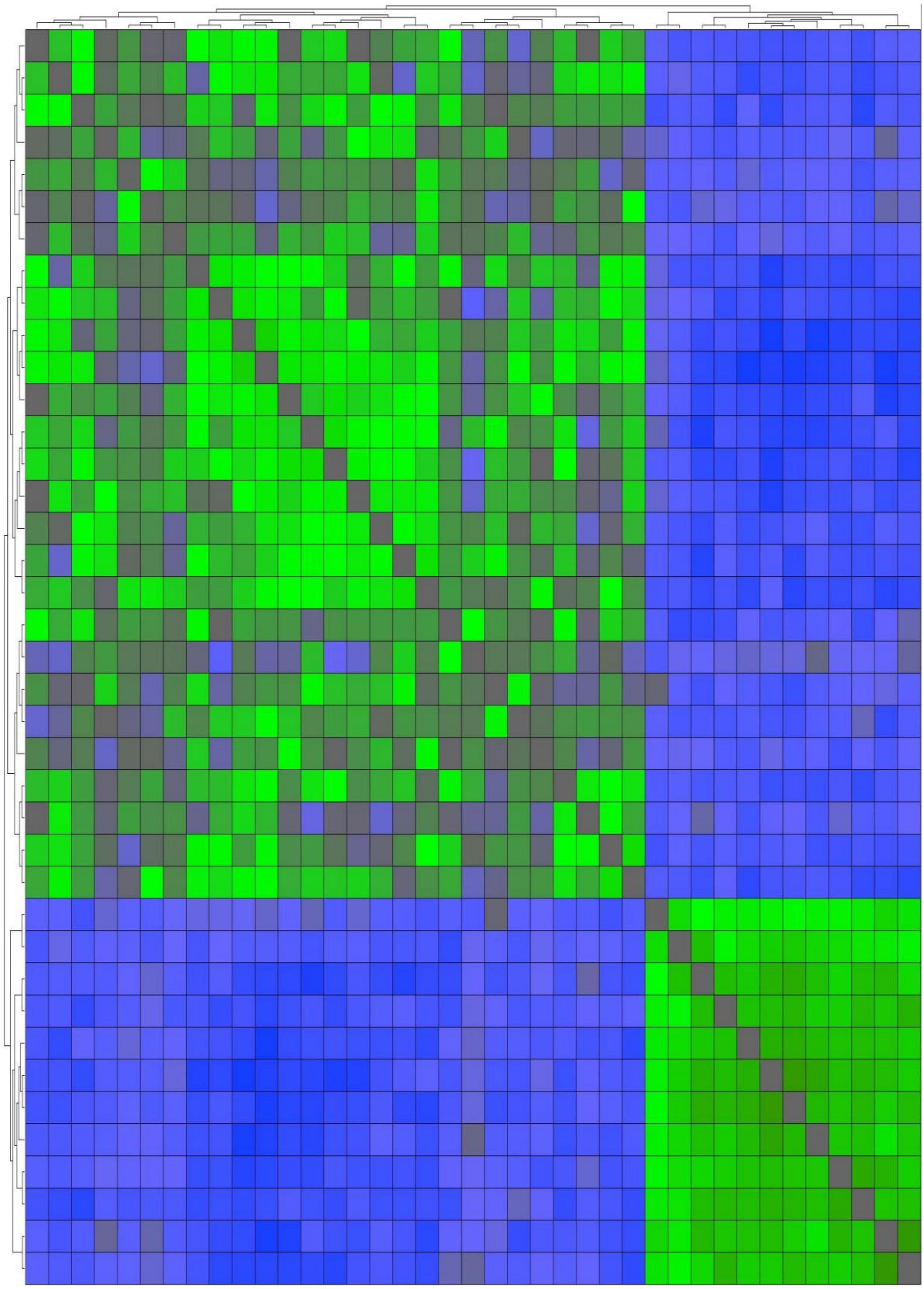

Figure S3. A correlation visualization with distance matrix displays the pairwise correlation between the 39 patients. Blue and green colors highlight the negative and positive correlation between samples. The genes found differently expressed were thereafter used to identify gene ontology terms (using the David Database for Gene Ontology) that were overrepresented among the genes differently expressed。

Table S1. A comparison of patients with and without dual AML cell populations; studies of molecular genetics, differentiation and pathway activation profiles. The two subsets do not differ with regard to frequencies of mutations, expression of differentiation Markers or level of PI3K-Akt-mTOR Activation.

**Additional Analysis of AML-Associated Mutations**		
**Classification of Mutations and Mutated Genes**[1]	**Dual Population**	**Single Population**
NPM1	28 %	32 %
Activated signaling<br>Flt3-ITD, Flt3-TKD, NRAS, KRAS, JAK2, PTPN11	67 %	64 %
Tumor Suppressor<br>TP53, WT1, CUX1, IKZF1, PHF6	33 %	24%
DNA methylation<br>DNMT3A, TET2, IDH1, IDH2	67 %	48 %
Chromatin modifiers<br>ASXL1, EZH2, GATA2	22 %	24 %
Myeloid transcription factors<br>RUNX1, CEBPA	28 %	24 %
Spliceosome<br>SRSF2, SF3B1, STAG2, RAD21, BCOR, BCORL1, CSF3R, ZRSR2	39 %	24 %
Cohesin<br>SMC1A	0	4 %
Others<br>NOTCH1, SETBP1	0	4%
**Molecular and Morphological Signs of Differentiation**		
**Expression of differentiation markers.** According to the WHO classification a minor subset of acute leukemia patients show expression of both myeloid and lymphoid markers by their leukemic cells, either as expression of both myeloid and lymphoid markers by the same cells or as separate myeloid and lymphoid subpopulations [2]. However, patients with and without dual populations in the PI3K-Akt-mTOR pathway activation analysis did not differ with regard to the expression of T cell (CD2, CD3, CD4, CD8) of B cell markers (CD19, CD20), and we could not detect any differences between the two patient subsets with regard to myeloid marker (DCD11b, CD11c, CD13, CD14, CD15, CD33), stem cell markers (CD34, CD117) or HLA-DR expression.<br>**Morphological signs of differentiation.** The FAB classification did not differ between the two subsets.		
**Activation Profile of The PI3K-AKT-mTor Pathway**		
The constitutive activation of mediators within the PI3K-Akt-mTOR pathway varies between patients [3]. Hence, we performed a clustering analysis to identify patients with similar pathway activation profiles, but the profiles did not differ between patients with and without detectable clonal heterogeneity.		

[1] The percentages of various mutations for patients without and with dual acute myeloid leukemia (AML) cell populations are presented. A total of 54 AML-associated mutations were investigated. The table lists only those mutations that were detected in the 43 unselected patients that were investigated, i.e. 18 patients with dual AML cell populations and 25 patients without clonal heterogeneity based on pathway activation analysis. [2] Referred in [1] [3] Referred in [2].

Table S2. The GO-term showing significant differences when comparing AML cell populations for patients with and without clonal heterogeneity; the comparison being based on analysis of Molecular function. The genes included in the analysis were identified by the comparison of 12 AML patient samples with and 27 samples without evidence for clonal heterogeneity when analyzing the PI3K-Akt-mTOR pathway. Two GO-terms were then identified based; G-protein coupled receptor activity (GO:0004930; *p*-value 0.00002; 36 genes included) and Olfactory receptor activity (GO:0004984; *p*-value 0.000024; 26 genes included). Several genes were included in both these terms; genes that did not overlap between these two terms are shaded in grey. Increased or decreased gene expression for patients with detectable subpopulations when analyzing constitutive PI3K-Akt-mTOR inhibition is indicated by upward arrows (↑) and downward arrows (↓), respectively.

**Molecular Function**

	GO:0004930 - G-protein coupled receptor activity; p-value 0.00002; 36 genes		GO:0004984 - Olfactory receptor activity; p-value 0.000024; 26 genes
↑	G protein-coupled receptor 132(GPR132)	↓	olfactory receptor family 1 subfamily D member 4 (gene/pseudogene)(OR1D4)
↑	G protein-coupled receptor 139(GPR139)	↓	olfactory receptor family 1 subfamily J member 2(OR1J2)
↓	G protein-coupled receptor 176(GPR176)	↓	olfactory receptor family 1 subfamily L member 8(OR1L8)
↑	G protein-coupled receptor 183(GPR183)	↑	olfactory receptor family 10 subfamily G member 3(OR10G3)
↑	G protein-coupled receptor 34(GPR34)	↓	olfactory receptor family 12 subfamily D member 3(OR12D3)
↓	G protein-coupled receptor class C group 5 member C(GPRC5C)	↓	olfactory receptor family 2 subfamily A member 4(OR2A4)
↓	G protein-coupled receptor class C group 6 member A(GPRC6A)	↓	olfactory receptor family 2 subfamily A member 42(OR2A42)
↓	cadherin EGF LAG seven-pass G-type receptor 1(CELSR1)	↓	olfactory receptor family 2 subfamily G member 3(OR2G3)
↓	frizzled class receptor 7(FZD7)	↓	olfactory receptor family 2 subfamily M member 4(OR2M4)
↓	olfactory receptor family 1 subfamily D member 4 (gene/pseudogene)(OR1D4)	↓	olfactory receptor family 2 subfamily T member 11 (gene/pseudogene)(OR2T11)
↓	olfactory receptor family 1 subfamily J member 2(OR1J2)	↓	olfactory receptor family 2 subfamily T member 12(OR2T12)
↓	olfactory receptor family 1 subfamily L member 8(OR1L8)	↑	olfactory receptor family 3 subfamily A member 3(OR3A3)
↑	olfactory receptor family 10 subfamily G member 3(OR10G3)	↑	olfactory receptor family 4 subfamily C member 16 (gene/pseudogene)(OR4C16)
↓	olfactory receptor family 12 subfamily D member 3(OR12D3)	↓	olfactory receptor family 4 subfamily C member 3(OR4C3)
↓	olfactory receptor family 2 subfamily A member 4(OR2A4)	↓	olfactory receptor family 4 subfamily C member 45(OR4C45)
↓	olfactory receptor family 2 subfamily A member 42(OR2A42)	↓	olfactory receptor family 4 subfamily F member 29(OR4F29)
↓	olfactory receptor family 2 subfamily G member 3(OR2G3)	↑	olfactory receptor family 5 subfamily AU member 1(OR5AU1)
↓	olfactory receptor family 2 subfamily M member 4(OR2M4)	↑	olfactory receptor family 5 subfamily H member 15(OR5H15)
↓	olfactory receptor family 2 subfamily T member 11 (gene/pseudogene)(OR2T11)	↑	olfactory receptor family 5 subfamily L member 1 (gene/pseudogene)(OR5L1)
↓	olfactory receptor family 2 subfamily T member 12(OR2T12)	↓	olfactory receptor family 51 subfamily G member 2(OR51G2)
↑	olfactory receptor family 3 subfamily A member 3(OR3A3)	↓	olfactory receptor family 52 subfamily L member 1(OR52L1)
↑	olfactory receptor family 4 subfamily C member 16 (gene/pseudogene)(OR4C16)	↑	olfactory receptor family 6 subfamily Y member 1(OR6Y1)
↓	olfactory receptor family 4 subfamily C member 3(OR4C3)	↓	olfactory receptor family 8 subfamily D member 4(OR8D4)
↓	olfactory receptor family 4 subfamily C member 45(OR4C45)	↓	olfactory receptor family 8 subfamily G member 2(OR8G2)
↓	olfactory receptor family 4 subfamily F member 29(OR4F29)	↑	olfactory receptor family 8 subfamily J member 1(OR8J1)
↑	olfactory receptor family 5 subfamily AU member 1(OR5AU1)	↑	olfactory receptor family 9 subfamily I member 1(OR9I1)

↑	olfactory receptor family 5 subfamily H member 15(OR5H15)
↑	olfactory receptor family 5 subfamily L member 1 (gene/pseudogene)(OR5L1)
↓	olfactory receptor family 51 subfamily G member 2(OR51G2)
↓	olfactory receptor family 52 subfamily L member 1(OR52L1)
↑	olfactory receptor family 6 subfamily Y member 1(OR6Y1)
↓	olfactory receptor family 8 subfamily D member 4(OR8D4)
↓	olfactory receptor family 8 subfamily G member 2(OR8G2)
↑	olfactory receptor family 8 subfamily J member 1(OR8J1)
↑	olfactory receptor family 9 subfamily I member 1(OR9I1)
↑	relaxin/insulin like family peptide receptor 1(RXFP1)

Table S3. The GO-term showing significant differences when comparing AML cell populations for patients with and without clonal heterogeneity; the comparison being based on analysis of Biological Processes. The genes included in the analysis were identified by the comparison of 12 AML patient samples with and 27 samples without evidence for clonal heterogeneity when analyzing the PI3K-Akt-mTOR pathway. Two GO-terms were then identified based; G-protein coupled receptor signaling pathway (GO:0007186; *p*-value 0.0000078; 43 genes) and Detection of chemical stimulus involved in sensory perception of smell (GO:0050911; *p*-value 0.000015; 26 genes). Several genes were included in both these terms; genes that did not overlap between these two terms are shaded in grey. Increased or decreased gene expression for patients with detectable subpopulations when analyzing constitutive PI3K-Akt-mTOR inhibition is indicated by upward arrows (↑) and downward arrows (↓), respectively.

**Biological Processes**			
	**GO:0007186 - G-protein coupled receptor signaling pathway; p-value 0.0000078; 43 genes**		**GO:0050911 - Detection of chemical stimulus involved in sensory perception of smell; p-value 0,000015; 26 genes**
↓	C-C motif chemokine ligand 15(CCL15)	↓	olfactory receptor family 1 subfamily D member 4 (gene/pseudogene)(OR1D4)
↑	G protein-coupled receptor 132(GPR132)	↓	olfactory receptor family 1 subfamily J member 2(OR1J2)
↑	G protein-coupled receptor 139(GPR139)	↓	olfactory receptor family 1 subfamily L member 8(OR1L8)
↓	G protein-coupled receptor 176(GPR176)	↑	olfactory receptor family 10 subfamily G member 3(OR10G3)
↑	G protein-coupled receptor 183(GPR183)	↓	olfactory receptor family 12 subfamily D member 3(OR12D3)
↑	G protein-coupled receptor 34(GPR34)	↓	olfactory receptor family 2 subfamily A member 4(OR2A4)
↓	G protein-coupled receptor class C group 5 member C(GPRC5C)	↓	olfactory receptor family 2 subfamily A member 42(OR2A42)
↓	G protein-coupled receptor class C group 6 member A(GPRC6A)	↓	olfactory receptor family 2 subfamily G member 3(OR2G3)
	NLR family pyrin domain containing 6(NLRP6)	↓	olfactory receptor family 2 subfamily M member 4(OR2M4)

↓	apolipoprotein C3(APOC3)	↓	olfactory receptor family 2 subfamily T member 11 (gene/pseudogene)(OR2T11)
↓	cadherin EGF LAG seven-pass G-type receptor 1(CELSR1)	↓	olfactory receptor family 2 subfamily T member 12(OR2T12)
↓	frizzled class receptor 7(FZD7)	↑	olfactory receptor family 3 subfamily A member 3(OR3A3)
↑	natriuretic peptide receptor 1(NPR1)	↑	olfactory receptor family 4 subfamily C member 16 (gene/pseudogene)(OR4C16)
↓	olfactory receptor family 1 subfamily D member 4 (gene/pseudogene)(OR1D4)	↓	olfactory receptor family 4 subfamily C member 3(OR4C3)
↓	olfactory receptor family 1 subfamily J member 2(OR1J2)	↓	olfactory receptor family 4 subfamily C member 45(OR4C45)
↓	olfactory receptor family 1 subfamily L member 8(OR1L8)	↓	olfactory receptor family 4 subfamily F member 29(OR4F29)
↑	olfactory receptor family 10 subfamily G member 3(OR10G3)	↑	olfactory receptor family 5 subfamily AU member 1(OR5AU1)
↓	olfactory receptor family 12 subfamily D member 3(OR12D3)	↑	olfactory receptor family 5 subfamily H member 15(OR5H15)
↓	olfactory receptor family 2 subfamily A member 4(OR2A4)	↑	olfactory receptor family 5 subfamily L member 1 (gene/pseudogene)(OR5L1)
↓	olfactory receptor family 2 subfamily A member 42(OR2A42)	↓	olfactory receptor family 51 subfamily G member 2(OR51G2)
	olfactory receptor family 2 subfamily G member 3(OR2G3)	↓	olfactory receptor family 52 subfamily L member 1(OR52L1)
↓	olfactory receptor family 2 subfamily M member 4(OR2M4)	↑	olfactory receptor family 6 subfamily Y member 1(OR6Y1)
↓	olfactory receptor family 2 subfamily T member 11 (gene/pseudogene)(OR2T11)	↓	olfactory receptor family 8 subfamily D member 4(OR8D4)
↓	olfactory receptor family 2 subfamily T member 12(OR2T12)	↓	olfactory receptor family 8 subfamily G member 2(OR8G2)
↑	olfactory receptor family 3 subfamily A member 3(OR3A3)	↑	olfactory receptor family 8 subfamily J member 1(OR8J1)
↑	olfactory receptor family 4 subfamily C member 16 (gene/pseudogene)(OR4C16)	↑	olfactory receptor family 9 subfamily I member 1(OR9I1)
↓	olfactory receptor family 4 subfamily C member 3(OR4C3)		
↓	olfactory receptor family 4 subfamily C member 45(OR4C45)		
↓	olfactory receptor family 4 subfamily F member 29(OR4F29)		
↑	olfactory receptor family 5 subfamily AU member 1(OR5AU1)		
↑	olfactory receptor family 5 subfamily H member 15(OR5H15)		
↑	olfactory receptor family 5 subfamily L member 1 (gene/pseudogene)(OR5L1)		
↓	olfactory receptor family 51 subfamily G member 2(OR51G2)		
↓	olfactory receptor family 52 subfamily L member 1(OR52L1)		
↑	olfactory receptor family 6 subfamily Y member 1(OR6Y1)		

↓	olfactory receptor family 8 subfamily D member 4(OR8D4)
↓	olfactory receptor family 8 subfamily G member 2(OR8G2)
↑	olfactory receptor family 8 subfamily J member 1(OR8J1)
↑	olfactory receptor family 9 subfamily I member 1(OR9I1)
↑	platelet derived growth factor receptor like(PDGFRL)
↑	prostaglandin E receptor 1(PTGER1)
↓	regulator of G-protein signaling 13(RGS13)
↑	vomeronasal 1 receptor 5 (gene/pseudogene)(VN1R5)

Table S4. Monoclonal antibodies used in the flow-cytometric studies.

Antibody	Manufacturer	Specificity
Alexa Fluor® 647 Mouse anti-PTEN	BD Biosciences	Recognizes PTEN, regardless of phosphorylation status
Alexa Fluor® 647 Mouse anti-PDPK1 (pS241)	BD Biosciences	Recognizes the phosphorylated S241 in the activation loop of human PDPK1
Alexa Fluor® 647 Mouse anti-PKCα	BD Biosciences	Recognizes PKCα , regardless of phosphorylation status
Alexa Fluor® 647 Mouse anti-PKCα (pT497)	BD Biosciences	Recognizes the phosphorylated T497 in the kinase domain of human PKC a
PE Mouse anti-Akt1	BD Biosciences	Recognizes Akt1, regardless of phosphorylation status
Alexa Fluor® 647 Mouse anti-Akt (pS473)	BD Biosciences	Recognizes Akt phosphorylated at S473
PE Mouse Anti-Akt (pT308)	BD Biosciences	Recognizes Akt phosphorylated at T308
Anti-RHEB antibody	Abcam	Specifically detects Rheb, regardless of phosphorylation status
Anti-Tuberin antibody	Abcam	Specifically detects Tuberin/TSC2
mTOR (7C10) Rabbit mAb (Alexa Fluor® 647 Conjugate)	Cell Signaling Technology	Detects endogenous levels of total mTOR protein
PE Mouse Anti-mTOR (pS2448)	BD Biosciences	Recognizes mTOR that is phosphorylated at S2448
Anti-Raptor antibody	Abcam	Specifically detects raptor, regardless of phosphorylation status
Anti-FKBP38 antibody	Abcam	Specifically detects FKBP38, regardless of phosphorylation status
Alexa Fluor® 647 Mouse anti-4EBP1 (pT36/pT45)	BD Biosciences	Recognizes the phosphorylated T36 and T45 of activated human 4EBP1
Alexa Fluor® 647 Mouse anti-elF4E (pS209)	BD Biosciences	Recognizes the phosphorylated S209 (pS209) of eIF4E
Alexa Fluor® 647 Mouse anti-S6 (pS244)	BD Biosciences	Specifically detects the S6 ribosomal protein phosphorylated at S244
PE Mouse anti-S6 (pS240)	BD Biosciences	Specifically detects the S6 ribosomal protein phosphorylated at S240
V450 Mouse anti-S6 (pS235/pS236)	BD Biosciences	Specifically detects the S6 ribosomal protein phosphorylated at S235 and S236

Table S5. The complete gene list from patients with dual AML cell populations. We compared the global gene expression profiles for 12 AML samples with and 27 samples without dual AML cell populations. A feature subset selection (FSS) analysis was performed for identification of the most discriminative genes between the two groups, and 1209 genes were then identified (i.e., p-value <0.05).

Gene ID	Symbol	Search Key	Illumina (ILM)-gene	P-value
127099	LOC127099	XM_060328.1	LOC127099	5.4429588162369E-5
100133469	LOC100133469	XM_001724642.1	LOC100133469	1.7669808120755886E-4
70	ACTC1	NM_005159.3	ACTC1	1.8889853780927157E-4
441762	LOC441762	XR_000268.2	LOC441762	1.9046862375469861E-4
29798	C2orf27A	NM_013310.3	C2ORF27A	2.295635289463627E-4
256302	C17orf103	NM_152914.1	C17ORF103	2.488848990283393E-4
100132215	LOC100132215	XM_001725462.1	LOC100132215	3.06887290218517E-4
100134277	LOC100134277	XM_001715852.1	LOC100134277	5.030423315792432E-4
402562	LOC402562	XM_939955.1	LOC402562	5.296271780355028E-4
92293	TMEM132C	XM_941994.1	TMEM132C	6.06081218427997E-4
100134261	LOC100134261	XM_001720725.1	LOC100134261	8.150840426564979E-4
125704	C18orf51	XM_945013.1	C18ORF51	8.157639850416456E-4
		Hs.582068	HS.582068	0.0010039973861543901
2857	GPR34	NM_001033513.1	GPR34	0.0011522345386179183
5602	MAPK10	NM_138980.1	MAPK10	0.0012104478969770386
1390	CREM	NM_001881.2	CREM	0.0012766444909731805
23213	SULF1	NM_015170.1	SULF1	0.0013817061034787775
127845	GOLT1A	NM_198447.1	GOLT1A	0.0014992835786023498
4143	MAT1A	NM_000429.2	MAT1A	0.00159068162706494
766	CA7	NM_005182.2	CA7	0.001644218788577064
647645	LOC647645	XM_942915.1	LOC647645	0.0019364782100578552
54801	HAUS6	NM_017645.3	HAUS6	0.0021784295379641723
64897	C12orf43	NM_022895.1	C12ORF43	0.0022888948062138475
645333	LOC645333	XM_932822.1	LOC645333	0.00229841711268192
100129122	LOC100129122	XM_001714405.1	LOC100129122	0.002358840674516944
650686	LOC650686	XM_001714190.1	LOC650686	0.002402213696133287
100130453	LOC100130453	XM_001717928.1	LOC100130453	0.0024686861240564824
728534	LOC728534	XM_496481.2	LOC728534	0.0024810022011861103
		Hs.544400	HS.544400	0.002570670446831798
		Hs.90866	HS.90866	0.002622155947935263
9578	CDC42BPB	NM_006035.2	CDC42BPB	0.0026464265370225127
390561	LOC390561	XM_931039.1	LOC390561	0.0030045102560638933
126006	PCP2	NM_174895.1	PCP2	0.003152601601927658
9994	CASP8AP2	NM_012115.2	CASP8AP2	0.003163468273950473
2266	FGG	NM_000509.4	FGG	0.003165510367807606
647307	LOC647307	XR_039752.1	LOC647307	0.0031975215144755143
100134549	LOC100134549	XM_001724842.1	LOC100134549	0.0032950797679523972
6812	STXBP1	NM_001032221.1	STXBP1	0.003339918394712154
643441	LOC643441	XM_933117.1	LOC643441	0.003445040137023792
1004	CDH6	NM_004932.2	CDH6	0.003606641166778987

646201	LOC646201	XR_038827.1	LOC646201	0.003735695619295815
124274	GPR139	NM_001002911.1	GPR139	0.0038673555386653483
		Hs.541889	HS.541889	0.003928735303692297
51557	LGSN	NM_016571.1	LGSN	0.00396746217456975
100129027	LOC100129027	XM_001726404.1	LOC100129027	0.004310105288322697
64241	ABCG8	NM_022437.2	ABCG8	0.004340420679539069
200576	PIP5K3	NM_152671.2	PIP5K3	0.004391969194981006
284359	IZUMO1	NM_182575.1	IZUMO1	0.004395986667519091
72	ACTG2	NM_001615.3	ACTG2	0.004571774525431297
		Hs.308351	HS.308351	0.004593524046106714
441873	LOC441873	XM_497653.2	LOC441873	0.004867506422327089
9247	GCM2	NM_004752.1	GCM2	0.004942484701647254
		Hs.564111	HS.564111	0.004988788091118323
148203	LOC148203	XM_938387.1	LOC148203	0.005112562979819541
441177	LOC441177	NM_001013720.1	LOC441177	0.0051398015983046535
9217	VAPB	NM_004738.3	VAPB	0.005175783078796158
391112	OR6Y1	NM_001005189.1	OR6Y1	0.0052210678682481125
338785	KRT79	NM_175834.2	KRT79	0.005267242199597024
10675	CSPG5	NM_006574.2	CSPG5	0.005338716851169746
4837	NNMT	NM_006169.2	NNMT	0.005340018415067846
731605	LOC731605	XM_001732887.1	LOC731605	0.0053742561572059306
158062	LCN6	NM_198946.2	LCN6	0.00551050561678674
		Hs.550619	HS.550619	0.005615448366994092
5903	RANBP2	NM_006267.3	RANBP2	0.005944117799023746
100134560	LOC100134560	XM_001718995.1	LOC100134560	0.00603271039077236
81575	APOLD1	NM_030817.1	APOLD1	0.0061665469488665905
55607	PPP1R9A	XM_934519.1	PPP1R9A	0.006209093089838259
51233	C22orf43	NM_016449.2	C22ORF43	0.006251254964463802
729731	LOC729731	XM_001131140.1	LOC729731	0.006443489149451054
641746	LOC641746	XR_036993.1	LOC641746	0.0064734119737271095
401399	LOC401399	XM_938040.1	LOC401399	0.006503933510587002
91445	RNF185	NM_152267.2	RNF185	0.006643879293476746
343069	HNRNPCL1	NM_001013631.1	HNRNPCL1	0.00668848680455546
653342	LOC653342	XM_926986.1	LOC653342	0.006704451522523867
		Hs.130971	HS.130971	0.006835062263307346
90326	THAP3	NM_138350.2	THAP3	0.006845201780484901
406993	MIR211	NR_029624.1	MIR211	0.007000712087657615
27253	PCDH17	NM_014459.2	PCDH17	0.007157587685755881
317705	VN1R5	NM_173858.1	VN1R5	0.007308537801323615
642698	LOC642698	XM_926147.1	LOC642698	0.007500827909900659
647591	LOC647591	XM_936642.1	LOC647591	0.00768313187287398
100129999	LOC100129999	XM_001723251.1	LOC100129999	0.007978078516773852
652874	LOC652874	XM_942590.1	LOC652874	0.008058888114695309
100133191	LOC100133191	XM_001720224.1	LOC100133191	0.008172412960470546
6792	CDKL5	NM_003159.2	CDKL5	0.008262129917024259
387267	KRTAP5-4	NM_001012709.1	KRTAP5-4	0.00833750183090909

400941	FLJ42418	NM_001001695.1	FLJ42418	0.008346757855315017
57611	ISLR2	NM_020851.1	ISLR2	0.008393630774620991
23269	MGA	NM_001080541.1	MGA	0.008679894079816232
85376	RIMBP3	NM_015672.1	RIMBP3	0.008898642569320844
284323	ZNF780A	NM_001010880.1	ZNF780A	0.008978682929154574
653539	LOC653539	XM_934566.1	LOC653539	0.009019103245916179
386681	KRTAP10-8	NM_198695.1	KRTAP10-8	0.009021269079951547
3800	KIF5C	NM_004522.1	KIF5C	0.009118444043063216
652882	LOC652882	XM_942599.1	LOC652882	0.009171572198060862
641955	LOC641955	XM_935721.1	LOC641955	0.0093460168089686
3620	IDO1	NM_002164.4	IDO1	0.009363909447106255
378884	NHLRC1	NM_198586.2	NHLRC1	0.0094580006051593
652737	LOC652737	XM_001718112.1	LOC652737	0.009546427444424247
54778	RNF111	NM_017610.6	RNF111	0.00968125336763508
100129083	LOC100129083	XM_001724757.1	LOC100129083	0.009689938019977624
51364	ZMYND10	NM_015896.2	ZMYND10	0.009907589492135787
		Hs.542544	HS.542544	0.009956858921022441
649909	LOC649909	XM_943982.1	LOC649909	0.010006376036609718
1325	CORT	NM_001302.3	CORT	0.010217548361631685
		Hs.563373	HS.563373	0.010407261517864428
59350	RXFP1	NM_021634.1	RXFP1	0.010545283382671588
7321	UBE2D1	NM_003338.3	UBE2D1	0.010566544072874764
84083	ZRANB3	NM_032143.2	ZRANB3	0.010841513823416579
158763	FLJ30058	NM_144967.2	FLJ30058	0.010930058649637631
10537	UBD	NM_006398.2	UBD	0.011172926856343891
54462	FAM190B	NM_018999.2	FAM190B	0.011271251153973216
650154	LOC650154	XM_944071.1	LOC650154	0.01129310117726207
11218	DDX20	NM_007204.3	DDX20	0.011344211025161022
644116	LOC644116	XM_933284.1	LOC644116	0.011369913354189182
4103	MAGEA4	NM_001011550.1	MAGEA4	0.011459719768653004
8085	MLL2	NM_003482.1	MLL2	0.011495307726533902
3709	ITPR2	NM_002223.1	ITPR2	0.011520969086767587
340481	ZDHHC21	NM_178566.2	ZDHHC21	0.011700678983183417
441332	FAM90A12	XM_496961.2	FAM90A12	0.011926926139427045
		Hs.27048	HS.27048	0.012139723893189567
100132839	LOC100132839	XM_001725950.1	LOC100132839	0.012251193131297656
643338	LOC643338	XM_927997.1	LOC643338	0.012309378127665593
		Hs.522935	HS.522935	0.012366037463090873
100313774	MIR302E	NR_031683.1	MIR302E	0.012466799678927403
		Hs.560698	HS.560698	0.012548076535386902
131831	FAM194A	NM_152394.2	FAM194A	0.012617196044206526
30818	KCNIP3	NM_013434.3	KCNIP3	0.012677315030705984
285311	C3orf56	NM_001007534.1	C3ORF56	0.01269820706499609
402381	SOHLH1	NM_001012415.1	SOHLH1	0.012733956612552355
643038	LOC643038	XM_926407.1	LOC643038	0.012838978911910548
		Hs.542481	HS.542481	0.013035512837795495

100302274	MIR1178	NR_031589.1	MIR1178	0.01305870627028648
		Hs.538176	HS.538176	0.013288378164829525
728747	LOC728747	XM_928873.1	LOC728747	0.013678378485791646
		Hs.565086	HS.565086	0.013681184497987222
9249	DHRS3	NM_004753.4	DHRS3	0.013702070752348021
100129808	LOC100129808	XR_039762.1	LOC100129808	0.013741295789919082
100133577	LOC100133577	XM_001716788.1	LOC100133577	0.013790216364984282
649991	LOC649991	XM_939078.1	LOC649991	0.01379355474644613
	ERCC-00098	ERCC-00098	ERCC-00098	0.013910035983225006
100134057	LOC100134057	XM_001718124.1	LOC100134057	0.01407780501664926
283687	C15orf37	NM_175898.2	C15ORF37	0.014124185960867468
3626	INHBC	NM_005538.2	INHBC	0.014353200725974444
644419	LOC644419	XM_932175.1	LOC644419	0.014357581612702485
2255	FGF10	NM_004465.1	FGF10	0.014382194667132114
100129583	FAM47E	NM_001136570.1	FAM47E	0.014423022504089493
		Hs.523127	HS.523127	0.01443578466046612
441956	LOC441956	XM_498859.2	LOC441956	0.01450209839706341
415	ARSE	NM_000047.1	ARSE	0.014587512708243683
406905	MIR1-2	NR_029662.1	MIR1-2	0.014600747058617292
9366	RAB9P1	NR_000039.1	RAB9P1	0.014848160505048023
6853	SYN1	NM_006950.2	SYN1	0.01507960250070801
727944	LOC727944	Hs.444961	LOC727944	0.015091515444061319
202459	LOC202459	NM_145303.1	LOC202459	0.015143355361588198
150244	FLJ31568	NM_152509.1	FLJ31568	0.015402338394228391
389633	LOC389633	XM_372030.4	LOC389633	0.015490223100747054
100132117	LOC100132117	XM_001725240.1	LOC100132117	0.015580093268710318
391670	LOC391670	XR_019025.1	LOC391670	0.015722796465197728
400156	RPS26L	NR_002225.2	RPS26L	0.01576965248812111
266697	POM121L4P	NR_024592.1	POM121L4P	0.01579271983878519
		Hs.560984	HS.560984	0.015800344711278804
254173	TTLL10	NM_153254.1	TTLL10	0.015812652787782506
6326	SCN2A	NM_021007.2	SCN2A	0.015940809007239015
653275	LOC653275	XM_932990.1	LOC653275	0.016001770436982435
		Hs.259386	HS.259386	0.016004345473892765
650975	LOC650975	XM_940080.1	LOC650975	0.016029588990556298
652258	LOC652258	XM_941668.1	LOC652258	0.01608013134347099
63974	NEUROD6	NM_022728.2	NEUROD6	0.016150373017089045
285643	KIF4B	NM_001099293.1	KIF4B	0.016248333616922687
		Hs.159049	HS.159049	0.016366558208216923
653424	LOC653424	XM_932020.1	LOC653424	0.01637344608415435
646269	LOC646269	XR_019325.2	LOC646269	0.016470839817159313
		Hs.390407	HS.390407	0.016512944520498406
8601	RGS20	NM_170587.1	RGS20	0.016542861227450488
51430	C1orf9	NM_014283.2	C1ORF9	0.01685998003402229
652798	LOC652798	XM_942459.1	LOC652798	0.016879126997924807
164684	WBP2NL	NM_152613.1	WBP2NL	0.016883078047378892

728603	FRMPD2L2	XM_931737.1	FRMPD2L2	0.016916786673588584
644710	LOC644710	XM_932320.1	LOC644710	0.017266395442179817
116966	WDR17	NM_170710.3	WDR17	0.017375785759740654
22801	ITGA11	NM_012211.3	ITGA11	0.01749005821141641
100133718	LOC100133718	XM_001713866.1	LOC100133718	0.01757902065841129
64493	C16orf10	NR_024121.1	C16ORF10	0.017662612203510786
653140	LOC653140	XM_931412.1	LOC653140	0.017685876567057807
375567	VWC2	NM_198570.1	VWC2	0.017809725623884182
100134592	LOC100134592	XR_039069.1	LOC100134592	0.01784849213198316
100133431	LOC100133431	XM_001713711.1	LOC100133431	0.018033690902546494
390507	LOC390507	XM_941221.1	LOC390507	0.018059490429512676
791115	PWRN2	NR_026647.1	PWRN2	0.01809236729739171
652128	LOC652128	XM_941465.1	LOC652128	0.018097957782505475
100302139	MIR1537	NR_031718.1	MIR1537	0.01813555205320322
		Hs.577082	HS.577082	0.018400209049827254
5176	SERPINF1	NM_002615.4	SERPINF1	0.018577222684230937
350	APOH	NM_000042.1	APOH	0.018690631761723663
10551	AGR2	NM_006408.3	AGR2	0.018848439149668654
323	APBB2	NM_173075.3	APBB2	0.01892954217116504
653419	LOC653419	XM_934475.1	LOC653419	0.018976474616645995
147650	LOC147650	NM_207324.1	LOC147650	0.018986756554713668
11096	ADAMTS5	NM_007038.2	ADAMTS5	0.01913768724155538
150368	FAM109B	NM_001002034.1	FAM109B	0.019167605385620224
406887	MIRLET7E	NR_029482.1	MIRLET7E	0.019214688371754666
64506	CPEB1	NM_030594.3	CPEB1	0.01924155328879387
		Hs.131656	HS.131656	0.01967564250413684
644090	LOC644090	XM_945887.1	LOC644090	0.019790031059694815
652071	LOC652071	XM_930113.1	LOC652071	0.019830966384617423
641852	LOC641852	XM_935593.1	LOC641852	0.019928268073840807
85360	SYDE1	NM_033025.4	SYDE1	0.020251423488305664
401101	LOC401101	XM_379234.3	LOC401101	0.020290327540105495
4008	LMO7	NM_005358.3	LMO7	0.02032681405922698
246213	SLC17A8	NM_139319.1	SLC17A8	0.02040938433833191
		Hs.450953	HS.450953	0.020425896992517793
100133118	LOC100133118	XM_001719352.1	LOC100133118	0.020561847635159287
3913	LAMB2	NM_002292.2	LAMB2	0.020727864885455943
		Hs.523508	HS.523508	0.02072946020971049
5345	SERPINF2	NM_000934.1	SERPINF2	0.02075928219921853
22844	FRMPD1	NM_014907.1	FRMPD1	0.02094698274076949
		Hs.577845	HS.577845	0.020961537623100948
4666	NACA	NM_005594.2	NACA	0.02100119453837278
255426	RASGEF1C	NM_001031799.1	RASGEF1C	0.021191749833611283
255626	HIST1H2BA	NM_170610.2	HIST1H2BA	0.021376189511903815
650965	LOC650965	XM_944440.1	LOC650965	0.0214008969373685
643534	LOC643534	XM_936822.2	LOC643534	0.021559336520668215
140738	TMEM37	NM_183240.1	TMEM37	0.021599174420979573

148170	CDC42EP5	NM_145057.2	CDC42EP5	0.021608582355112586
8139	GAN	NM_022041.2	GAN	0.02170980675855108
642441	LOC642441	XM_930678.3	LOC642441	0.021736426354992913
139189	DGKK	NM_001013742.1	DGKK	0.021767396571142218
650180	LOC650180	XM_939263.1	LOC650180	0.02182579988548141
933	CD22	NM_001771.1	CD22	0.021869730561595586
442251	LOC442251	XM_498135.2	LOC442251	0.02187831310995123
644037	LOC644037	XR_017337.2	LOC644037	0.022220250277086427
337967	KRTAP6-2	NM_181604.1	KRTAP6-2	0.02222310695794649
644841	LOC644841	XM_927936.1	LOC644841	0.022422888467140092
		Hs.578887	HS.578887	0.02243599378753856
284541	CYP4A22	NM_001010969.2	CYP4A22	0.022461197895480303
		Hs.544737	HS.544737	0.022511129189707275
81555	YIPF5	NM_030799.6	YIPF5	0.022539229264113298
649501	LOC649501	XM_938580.1	LOC649501	0.022567620190517213
29933	GPR132	NM_013345.2	GPR132	0.022575922292977182
729732	LOC729732	XM_932827.1	LOC729732	0.022685263572146966
1946	EFNA5	NM_001962.1	EFNA5	0.022870521985390184
9957	HS3ST1	NM_005114.2	HS3ST1	0.023096833903532978
100131294	LOC100131294	XM_001718622.1	LOC100131294	0.02335883403484874
219437	OR5L1	NM_001004738.1	OR5L1	0.023421551832223223
728753	FAM90A19	XM_001129368.2	FAM90A19	0.02348762306281699
83999	KREMEN1	NM_032045.3	KREMEN1	0.023749641111062317
55871	CBWD1	NM_018491.3	CBWD1	0.0237621427097727
81930	KIF18A	NM_031217.2	KIF18A	0.023886877809522717
730077	LOC730077	XM_929823.1	LOC730077	0.023982448377878662
55554	KLK15	NM_017509.2	KLK15	0.0240493655076375
27290	SPINK4	NM_014471.1	SPINK4	0.02408277086088165
349114	LOC349114	XR_040229.1	LOC349114	0.024269512235205234
100131223	LOC100131223	XR_039276.1	LOC100131223	0.02448826957591335
79750	ZNF385D	NM_024697.1	ZNF385D	0.02449260284148093
652608	LOC652608	XM_942140.1	LOC652608	0.024538555779626832
390445	OR5AU1	NM_001004731.1	OR5AU1	0.024666882109893585
1446	CSN1S1	NM_001890.1	CSN1S1	0.0247003630519056
574436	MIR485	NR_030160.1	MIR485	0.024719706991961146
653453	LOC653453	XM_933338.1	LOC653453	0.02494463989225702
85508	SCRT2	NM_033129.1	SCRT2	0.025139068866499865
645649	LOC645649	XM_928663.1	LOC645649	0.025223184090347517
171389	NLRP6	NM_138329.1	NLRP6	0.025317308419384465
133482	SLCO6A1	NM_173488.2	SLCO6A1	0.025329794343735376
		Hs.132448	HS.132448	0.02553378780006922
80818	ZNF436	NM_030634.1	ZNF436	0.0255551614087837
80031	SEMA6D	NM_024966.2	SEMA6D	0.025558717309230792
92340	C17orf72	NR_015354.2	C17ORF72	0.025633877058255945
		Hs.399823	HS.399823	0.025670833890299852
100133067	LOC100133067	XM_001725464.1	LOC100133067	0.02589841479039926

23262	HISPPD1	NM_015216.2	HISPPD1	0.025898595112993666
		Hs.537971	HS.537971	0.026063789227305127
54822	TRPM7	NM_017672.2	TRPM7	0.0261330076463797
100132088	LOC100132088	XR_037760.1	LOC100132088	0.026147330213606424
10846	PDE10A	NM_006661.1	PDE10A	0.02617993149900035
		Hs.494628	HS.494628	0.026203085945303402
100128328	LOC100128328	XM_001715053.1	LOC100128328	0.026391195653177747
284912	LOC284912	NM_203375.1	LOC284912	0.02645942324817142
100133959	LOC100133959	XM_001720421.1	LOC100133959	0.026649547761975846
5205	ATP8B1	NM_005603.2	ATP8B1	0.026998467102858623
		Hs.164254	HS.164254	0.02708058345318273
645870	LOC645870	XM_930479.1	LOC645870	0.02708664365272622
5681	PSKH1	NM_006742.1	PSKH1	0.027094411579846493
644264	LOC644264	XM_927442.1	LOC644264	0.027203704934803792
23414	ZFPM2	NM_012082.2	ZFPM2	0.027380423257296505
219954	OR9I1	NM_001005211.1	OR9I1	0.027405531858696788
100129461	LOC100129461	XM_001719437.1	LOC100129461	0.027424973554395375
54477	PLEKHA5	NM_019012.2	PLEKHA5	0.027462476844466694
29785	CYP2S1	NM_030622.5	CYP2S1	0.027469637841125567
57045	TWSG1	NM_020648.3	TWSG1	0.027547802917928584
10869	USP19	NM_006677.1	USP19	0.027715324591564602
100133911	LOC100133911	XM_001714502.1	LOC100133911	0.02782298055874275
100132029	LOC100132029	XM_001719312.1	LOC100132029	0.027832907304590242
651125	LOC651125	XM_940255.1	LOC651125	0.02787994837746972
257396	LOC257396	XM_001715443.1	LOC257396	0.027909105492990874
730389	LOC730389	XM_001726418.1	LOC730389	0.02802181380683962
649151	LOC649151	XM_944782.1	LOC649151	0.028035977164727922
57084	SLC17A6	NM_020346.1	SLC17A6	0.02808767449264285
10417	SPON2	NM_012445.1	SPON2	0.02809670267559562
729826	LOC729826	XM_932003.1	LOC729826	0.02810333996192765
138715	ARID3C	NM_001017363.1	ARID3C	0.028113477207784367
		Hs.543971	HS.543971	0.02817707262444036
400844	FLJ42133	NM_001001690.1	FLJ42133	0.02820580226917168
		Hs.444411	HS.444411	0.028238626580358715
401648	LOC401648	XR_038983.1	LOC401648	0.028314991818208114
650428	LOC650428	XM_939516.1	LOC650428	0.028702941205273995
100133896	LOC100133896	XM_001723682.1	LOC100133896	0.028715684876214117
401491	FLJ35024	NR_015375.1	FLJ35024	0.028811076040184593
221468	TMEM217	XM_929843.1	TMEM217	0.029022603350601032
11185	INMT	NM_006774.4	INMT	0.0291037479385151
100129159	LOC100129159	XM_001719666.1	LOC100129159	0.029374739907140807
389671	LOC389671	XM_374275.2	LOC389671	0.029484891395720376
154288	C6orf221	NM_001017361.2	C6ORF221	0.029593828182104785
647154	LOC647154	XM_934436.1	LOC647154	0.029829653041726342
386724	AMIGO3	NM_198722.1	AMIGO3	0.029951202277859936
89796	NAV1	NM_020443.2	NAV1	0.02999043524271502

56751	BARHL1	NM_020064.2	BARHL1	0.030022852681444435
133619	PRRC1	NM_130809.2	PRRC1	0.030187721912594043
64122	FN3K	NM_022158.2	FN3K	0.03021107536473889
56254	RNF20	NM_019592.5	RNF20	0.03033470528345383
8771	TNFRSF6B	NM_032945.2	TNFRSF6B	0.03035914723145836
8392	OR3A3	NM_012373.1	OR3A3	0.03053809988231804
		Hs.133261	HS.133261	0.030568099022101985
100131124	LOC100131124	XR_038480.1	LOC100131124	0.03061359552459085
655	BMP7	NM_001719.1	BMP7	0.030651295946450993
145447	ABHD12B	NM_181533.3	ABHD12B	0.030664450871831905
114902	C1QTNF5	NM_015645.2	C1QTNF5	0.0307441372846035
1272	CNTN1	NM_001843.2	CNTN1	0.030973923100136438
100134397	LOC100134397	XM_001716222.1	LOC100134397	0.03102925073273608
4747	NEFL	NM_006158.1	NEFL	0.031244890324453
51201	ZDHHC2	NM_016353.2	ZDHHC2	0.03130797889576718
645038	LOC645038	XM_932599.1	LOC645038	0.03134188336365769
100131507	LOC100131507	XR_038156.1	LOC100131507	0.031346082065930175
		Hs.195035	HS.195035	0.031634632331781154
654346	LGALS9C	NM_001040078.2	LGALS9C	0.031764887661290764
8563	THOC5	NM_001002878.1	THOC5	0.03191725864259395
100132705	LOC100132705	XM_001715205.1	LOC100132705	0.03192907747211482
55545	MSX2P1	NR_002307.1	MSX2P1	0.0319808871078602
219428	OR4C16	NM_001004701.1	OR4C16	0.03203493369424288
647247	LOC647247	XM_934568.1	LOC647247	0.032152819934808
56849	TCEAL7	NM_152278.1	TCEAL7	0.03222680725793716
57175	CORO1B	NM_020441.2	CORO1B	0.03222783672970617
641768	LOC641768	XM_935907.3	LOC641768	0.0322664317396529
197407	ZNF48	NM_152652.1	ZNF48	0.03229426087776541
100302163	MIR1278	NR_031691.1	MIR1278	0.032440391913297596
55679	LIMS2	NM_017980.2	LIMS2	0.032517058083104546
5834	PYGB	NM_002862.3	PYGB	0.032539078867889336
644934	LOC644934	XM_934677.1	LOC644934	0.0325617138291315
1302	COL11A2	NM_080681.1	COL11A2	0.03256917573410545
		Hs.156178	HS.156178	0.03263352286288794
725	C4BPB	NM_001017367.1	C4BPB	0.03290258407073522
3741	KCNA5	NM_002234.2	KCNA5	0.033000791233888896
441233	LOC441233	NM_001013724.1	LOC441233	0.03308686516536579
651008	LOC651008	XM_940120.1	LOC651008	0.03331768852877934
440243	LOC440243	XM_496041.3	LOC440243	0.033377254112434064
653876	LOC653876	XM_936221.1	LOC653876	0.033379693490334726
79677	SMC6	NM_024624.3	SMC6	0.03343033375558616
401957	LOC401957	XM_496379.2	LOC401957	0.03343759911919372
114815	SORCS1	NM_052918.3	SORCS1	0.03385617247917471
100128404	LOC100128404	XM_001713786.1	LOC100128404	0.03386855319301855
652286	LOC652286	XM_941705.1	LOC652286	0.033962860994464016
399706	LOC399706	NM_001010910.1	LOC399706	0.034007211184900424

1413	CRYBA4	NM_001886.1	CRYBA4	0.03401175055796066
441502	RPS26P11	NR_002309.1	RPS26P11	0.03409019967664746
		Hs.207074	HS.207074	0.034157092547502425
11281	POU6F2	NM_007252.2	POU6F2	0.0342334160199717
25903	OLFML2B	NM_015441.1	OLFML2B	0.03426761739469739
911	CD1C	NM_001765.1	CD1C	0.03437678703029185
201181	ZNF385C	NM_001013624.1	ZNF385C	0.03442394727482257
		Hs.61151	HS.61151	0.03450872404206222
		Hs.572889	HS.572889	0.034602714666213466
283824	LOC283824	XM_936476.1	LOC283824	0.03469855579099817
389320	C5orf48	NM_207408.1	C5ORF48	0.03480603097892593
643943	LOC643943	XM_934575.1	LOC643943	0.03492449423224515
84140	FLJ13305	NM_032180.1	FLJ13305	0.034924817386345224
653149	NBPF6	XM_926213.3	NBPF6	0.034948186249392776
83755	KRTAP4-12	NM_031854.2	KRTAP4-12	0.03495961113206711
644222	LOC644222	XM_932052.1	LOC644222	0.03502179995398587
403274	OR5H15	NM_001005515.1	OR5H15	0.03528865792443498
642302	LOC642302	XM_925847.1	LOC642302	0.03532679261216908
5757	PTMA	NM_002823.2	PTMA	0.03533048715758714
85449	KIAA1755	NM_001029864.1	KIAA1755	0.03550092188291261
280658	SSX7	NM_173358.2	SSX7	0.035527787941814014
		Hs.559234	HS.559234	0.035570714704618304
8894	EIF2S2	NM_003908.3	EIF2S2	0.03570589969817165
647123	LOC647123	XM_930141.1	LOC647123	0.035816525085092685
353140	LCE2C	NM_178429.2	LCE2C	0.035934579863750746
677801	SNORA14A	NR_002955.1	SNORA14A	0.0361854163552442
125058	TBC1D16	NM_019020.2	TBC1D16	0.03626812263100942
652469	LOC652469	XM_941921.1	LOC652469	0.0362927530207742
		Hs.66072	HS.66072	0.03637146426565126
7128	TNFAIP3	NM_006290.2	TNFAIP3	0.03639524499582533
84667	HES7	NM_032580.1	HES7	0.0364117990492306
644928	LOC644928	NM_001093732.1	LOC644928	0.036419987574674156
7809	BSND	NM_057176.2	BSND	0.03653091396146062
440792	LOC440792	XM_496493.2	LOC440792	0.03654208725894107
492303	LOC492303	NR_002830.1	LOC492303	0.03660139461851856
401281	FLJ27255	NM_207501.1	FLJ27255	0.03662507602211143
391767	LOC391767	XM_936313.1	LOC391767	0.036632788973511736
645141	LOC645141	XM_932673.1	LOC645141	0.036680323689545984
375607	C7orf52	NM_198571.1	C7ORF52	0.03678010633215606
400169	DKFZp451A211	NM_001003399.1	DKFZP451A211	0.037087843662292584
441257	LOC441257	NM_001023562.1	LOC441257	0.03748984854102287
5998	RGS3	NM_130795.2	RGS3	0.03750498381085232
30845	EHD3	NM_014600.1	EHD3	0.03752440328750746
128209	KLF17	NM_173484.2	KLF17	0.03753163799650583
86614	HSFY1	NM_001001871.1	HSFY1	0.03756517173064518
643717	LOC643717	XM_931762.1	LOC643717	0.03759956434879695

3185	HNRNPF	NM_004966.2	HNRNPF	0.03766736732960908
60482	SLC5A7	NM_021815.2	SLC5A7	0.03777752067761614
84775	ZNF607	NM_032689.3	ZNF607	0.037833110045205016
646982	LOC646982	XM_929953.1	LOC646982	0.037858020157582745
26533	OR10G3	NM_001005465.1	OR10G3	0.03787398889924781
100132994	LOC100132994	XM_001716545.1	LOC100132994	0.0381880201170454
51454	GULP1	NM_016315.2	GULP1	0.03823336785815221
147670	LOC147670	XM_938354.1	LOC147670	0.038271133361022
100133667	LOC100133667	XM_001716955.1	LOC100133667	0.038353586825590834
441377	LOC441377	XM_938599.1	LOC441377	0.038425188952630224
		Hs.576557	HS.576557	0.03845300174614561
27190	IL17B	NM_014443.2	IL17B	0.03851235934753757
1612	DAPK1	NM_004938.1	DAPK1	0.038513267889527844
406981	MIR19B2	NR_029491.1	MIR19B2	0.03865875848007942
100129489	LOC100129489	XM_001725742.1	LOC100129489	0.0388785449116513
389043	LOC389043	XM_374012.2	LOC389043	0.038894805019877444
2326	FMO1	NM_002021.1	FMO1	0.0389169991967331
643369	LOC643369	XM_926699.1	LOC643369	0.03894931459555177
646688	LOC646688	XR_038043.1	LOC646688	0.03904764703794127
407009	MIR224	NR_029638.1	MIR224	0.039111411385302795
9991	ROD1	NM_005156.4	ROD1	0.03912009402141927
55901	THSD1	NM_018676.2	THSD1	0.0391402794320996
387742	FAM99A	NM_001014374.1	FAM99A	0.039529403362823914
504188	LOC504188	NM_001013404.1	LOC504188	0.03960475777464169
55748	CNDP2	NM_018235.1	CNDP2	0.03969157665674203
641515	LOC641515	XR_041510.1	LOC641515	0.0397689232251305
80318	GKAP1	NM_025211.2	GKAP1	0.03986648588847759
389376	SFTA2	NM_205854.1	SFTA2	0.03999423728853999
648533	LOC648533	XM_937587.1	LOC648533	0.04031944639793983
10694	CCT8	NM_006585.2	CCT8	0.0403292788968682
6098	ROS1	NM_002944.2	ROS1	0.04033312843536948
6670	SP3	NM_003111.3	SP3	0.04042713037654128
1404	HAPLN1	NM_001884.2	HAPLN1	0.04048525841710001
4881	NPR1	NM_000906.2	NPR1	0.040502565677173176
649801	LOC649801	XM_938871.1	LOC649801	0.04050521442632797
650144	LOC650144	XM_939226.1	LOC650144	0.04054045691347192
4887	NPY2R	NM_000910.2	NPY2R	0.04079826941342508
60439	TTTY2	NR_001536.1	TTTY2	0.04092232641545795
652517	LOC652517	XM_941993.1	LOC652517	0.041006153896141075
		Hs.575322	HS.575322	0.04100760502736633
644421	LOC644421	XM_932176.1	LOC644421	0.04115702923750378
		Hs.567469	HS.567469	0.04119812979787987
644150	LOC644150	XM_933686.1	LOC644150	0.04121231842909383
26301	GBGT1	NM_021996.3	GBGT1	0.04133282944557231
56947	MFF	NM_020194.4	MFF	0.04134914870555508
124790	HEXIM2	NM_144608.1	HEXIM2	0.0414099130711993

4781	NFIB	NM_005596.1	NFIB	0.04142927296674448
652667	LOC652667	XM_942241.1	LOC652667	0.04145856823506089
		Hs.550320	HS.550320	0.041472016762589686
728353	LOC728353	XR_041035.1	LOC728353	0.04148634552345762
		Hs.538064	HS.538064	0.041536830424488996
100130648	LOC100130648	XM_001719568.1	LOC100130648	0.04156307760807585
100302138	MIR1292	NR_031699.1	MIR1292	0.041597253865857386
54842	MFSD6	NM_017694.2	MFSD6	0.04168319322226114
100129722	LOC100129722	XM_001723078.1	LOC100129722	0.04178887578344652
728897	LOC728897	XM_001714242.1	LOC728897	0.04179921046302966
		Hs.570505	HS.570505	0.04186242156445938
642828	LOC642828	XR_016385.2	LOC642828	0.04189952262354723
128876	FAM83C	NM_178468.2	FAM83C	0.0420309294383992
7350	UCP1	NM_021833.3	UCP1	0.04209019401793435
57703	CWC22	NM_020943.1	CWC22	0.042102050543425557
2162	F13A1	NM_000129.2	F13A1	0.042112733355483295
8463	TEAD2	NM_003598.1	TEAD2	0.04212361026047233
123624	AGBL1	Hs.569517	AGBL1	0.042251663296841206
51155	HN1	NM_016185.2	HN1	0.04254373110656518
654085	LOC654085	XM_942123.1	LOC654085	0.04257358154415917
		Hs.547985	HS.547985	0.04260022423480719
4780	NFE2L2	NM_006164.2	NFE2L2	0.04267011054111266
728612	LOC728612	XR_015601.1	LOC728612	0.0427617681836858
100131909	LOC100131909	XM_001722362.1	LOC100131909	0.04290454348425088
23284	LPHN3	NM_015236.3	LPHN3	0.04330834214616471
85443	DCLK3	NM_033403.1	DCLK3	0.043353620219200605
9840	KIAA0748	XM_934138.1	KIAA0748	0.04336354602898456
650646	LOC650646	XM_930416.1	LOC650646	0.04349481866826087
26168	SENP3	NM_015670.3	SENP3	0.043643046874244765
441655	LOC441655	XM_497366.2	LOC441655	0.043662954044794984
653051	LOC653051	XM_925795.1	LOC653051	0.04369705229509728
651465	LOC651465	XM_944690.1	LOC651465	0.04376632172718305
642712	LOC642712	XM_926777.1	LOC642712	0.043815420015324714
9154	SLC28A1	NM_201651.1	SLC28A1	0.043841747366748285
100128746	LOC100128746	XM_001726588.1	LOC100128746	0.043874905167078086
646566	LOC646566	XM_933706.1	LOC646566	0.04390382362985182
729356	LOC729356	XR_036863.1	LOC729356	0.04392491941625278
169834	LOC169834	XM_936297.1	LOC169834	0.04406591788604947
647836	LOC647836	XM_936893.1	LOC647836	0.044074300034243834
79158	GNPTAB	NM_024312.3	GNPTAB	0.044177209332562146
		Hs.551358	HS.551358	0.04429590120343183
55511	SAGE1	NM_018666.1	SAGE1	0.04454669580107023
114088	TRIM9	NM_052978.3	TRIM9	0.044713601155745256
1880	GPR183	NM_004951.4	GPR183	0.044766978044226774
58525	WIZ	NM_021241.2	WIZ	0.04487145485387742
		Hs.570535	HS.570535	0.0448985342130216

646040	LOC646040	XM_933354.1	LOC646040	0.044910321983449665
100130663	LOC100130663	XM_001719837.1	LOC100130663	0.0449171581004836
10156	RASA4	NM_006989.3	RASA4	0.0449172733642023
100129412	LOC100129412	XM_001724449.1	LOC100129412	0.04509846942017952
693142	MIR557	NR_030284.1	MIR557	0.04522478913460713
83898	KRTAP4-8	XM_927495.1	KRTAP4-8	0.04533959100938478
8440	NCK2	NM_003581.2	NCK2	0.045390805618902325
26582	DUX3	NM_012148.2	DUX3	0.045444015891660465
51338	MS4A4A	NM_148975.1	MS4A4A	0.045824373203041946
23090	ZNF423	NM_015069.2	ZNF423	0.0458380449604099
112399	EGLN3	NM_022073.2	EGLN3	0.04586641476579686
645450	LOC645450	XM_928482.1	LOC645450	0.04587792597520967
		Hs.565704	HS.565704	0.04604524295035083
727994	LOC727994	XM_001717645.1	LOC727994	0.04616846813419221
5157	PDGFRL	NM_006207.1	PDGFRL	0.046193739473114004
282969	C10orf125	NM_198472.1	C10ORF125	0.04621714785670731
647983	LOC647983	XM_937039.1	LOC647983	0.046354241561175194
79031	PDCL3	XM_929879.1	PDCL3	0.04641754842007101
100313938	MIR548G	NR_031662.1	MIR548G	0.04655543373008615
1611	DAP	NM_004394.1	DAP	0.04656125580018756
391241	LOC391241	XM_372864.3	LOC391241	0.046576699058196815
150921	TCF23	NM_175769.1	TCF23	0.04661232178619408
619554	MIR486	NR_030161.1	MIR486	0.046612985679481606
100132979	LOC100132979	XM_001723476.1	LOC100132979	0.046630454056350454
651362	LOC651362	XM_940505.1	LOC651362	0.04669258782808066
8125	ANP32A	NM_006305.2	ANP32A	0.046783736391782925
2887	GRB10	NM_005311.3	GRB10	0.046952512945653696
26235	FBXL4	NM_012160.3	FBXL4	0.04696753087023498
644714	LOC644714	XM_934514.1	LOC644714	0.04703281511457221
727910	LOC727910	XM_001718130.1	LOC727910	0.047078199178451074
		Hs.571403	HS.571403	0.04708841921734624
100134805	LOC100134805	XM_001719203.1	LOC100134805	0.04712513232533933
745	C11orf9	NM_013279.1	C11ORF9	0.04715254550434623
160492	IFLTD1	NM_152590.1	IFLTD1	0.04717322013598507
7015	TERT	NM_198253.2	TERT	0.04718613531279058
		Hs.545952	HS.545952	0.04722315518419996
8624	PSMG1	NM_203433.1	PSMG1	0.04737253754741517
100129271	C1orf68	NM_001024679.2	C1ORF68	0.04737332589684549
219477	OR8J1	NM_001005205.1	OR8J1	0.04737755389151637
3872	KRT17	NM_000422.1	KRT17	0.047425982292438607
643493	LOC643493	XM_931578.1	LOC643493	0.047431793499921646
2710	GK	NM_000167.3	GK	0.04747795359305717
8528	DDO	NM_003649.2	DDO	0.04759894764627622
54868	TMEM104	NM_017728.2	TMEM104	0.04763931569476485
64786	TBC1D15	NM_022771.3	TBC1D15	0.04776252741607036
100302129	MIR1915	NR_031736.1	MIR1915	0.04780680859532939

729475	RAD51AP2	Hs.515802	RAD51AP2	0.04782097794386552
		Hs.544828	HS.544828	0.048024235371097825
		Hs.580655	HS.580655	0.04817920321578044
652845	LOC652845	XM_942543.1	LOC652845	0.04829554628170876
	ERCC-00111	ERCC-00111	ERCC-00111	0.04833840947152641
		Hs.260074	HS.260074	0.04835079602446493
3231	HOXD1	NM_024501.1	HOXD1	0.048401910255265405
64377	CHST8	NM_022467.3	CHST8	0.04858975405102501
		Hs.560387	HS.560387	0.04860858415283664
100134121	LOC100134121	XM_001714675.1	LOC100134121	0.04867882754541401
10053	AP1M2	NM_005498.3	AP1M2	0.048839436915587696
100132649	LOC100132649	XM_001718045.1	LOC100132649	0.04887013981366635
6169	RPL38	NM_001035258.1	RPL38	0.04897774040010492
652512	LOC652512	XM_941983.1	LOC652512	0.04917845930977059
8819	SAP30	NM_003864.1	SAP30	0.04921232319667151
57507	ZNF608	NM_020747.1	ZNF608	0.0493426107426408
2658	GDF2	NM_016204.1	GDF2	0.04942968746423412

Table S5. The complete gene list from patients without dual AML cell populations. We compared the global gene expression profiles for 12 AML samples with and 27 samples without dual AML cell populations. A feature subset selection (FSS) analysis was performed for identification of the most discriminative genes between the two groups, and 1209 genes were then identified (i.e., p-value <0.05).

**Gene ID**	**Symbol**	**Search Key**	**Illumina (ILM)-gene**	**P-value**
642528	LOC642528	XM_926022.1	LOC642528	1.817595639495555E-4
100130508	LOC100130508	XM_001716255.1	LOC100130508	4.447421112487998E-4
		Hs.567094	HS.567094	6.800822576455169E-4
643857	LOC643857	XM_927130.1	LOC643857	7.808779365329758E-4
653111	LOC653111	XM_926073.2	LOC653111	8.070077698455909E-4
401498	LOC401498	NM_212558.1	LOC401498	8.296802641247719E-4
		Hs.313888	HS.313888	9.294829916806852E-4
203414	CXorf24	XM_926772.1	CXORF24	9.756606904733263E-4
		Hs.535372	HS.535372	0.001000817614127855
23555	TSPAN15	NM_012339.3	TSPAN15	0.001079624881794884
642553	LOC642553	XM_926041.1	LOC642553	0.0011128384035927249
254225	RNF169	XM_940188.1	RNF169	0.0011286947961539869
284293	HMSD	NM_001123366.1	HMSD	0.0012888164659033512
9182	RASSF9	NM_005447.2	RASSF9	0.0013483219634245417
574446	MIR511-2	NR_030168.1	MIR511-2	0.0013825423617592363
100134461	LOC100134461	XR_039702.1	LOC100134461	0.0013886851670043363
643464	LOC643464	XM_931563.1	LOC643464	0.001403925102163556
		Hs.561144	HS.561144	0.0014203934909458207
127150	LOC127150	XM_497717.2	LOC127150	0.0014787996671713956
100134368	LOC100134368	NR_024453.1	LOC100134368	0.0014831756022624565
644451	LOC644451	XR_037207.1	LOC644451	0.0017513812633183805
100133232	LOC100133232	XM_001720154.1	LOC100133232	0.0018682046476840581
642608	LOC642608	XM_930949.1	LOC642608	0.0018893903351726647

		Hs.563292	HS.563292	0.0019857574463121937
649189	LOC649189	XM_943611.1	LOC649189	0.0020192891414445874
388572	LOC388572	XM_001722169.1	LOC388572	0.0020738138287287465
646334	LOC646334	XM_929268.1	LOC646334	0.0020862534372304044
		Hs.232517	HS.232517	0.0021399762832749383
654209	LOC654209	XM_941064.1	LOC654209	0.0022286393784698762
288	ANK3	NM_020987.2	ANK3	0.0022312829691701246
344148	NCKAP5	NM_207363.2	NCKAP5	0.002265105586918537
653796	LOC653796	XM_929960.1	LOC653796	0.0023010467263336162
84931	FLJ14816	XR_017947.2	FLJ14816	0.002610085693546829
647219	LOC647219	XM_930260.1	LOC647219	0.0026196262039302792
652355	LOC652355	XM_939668.1	LOC652355	0.00274036261086637
1583	CYP11A1	NM_000781.1	CYP11A1	0.002750373015735045
130	ADH6	NM_000672.2	ADH6	0.0028473581005427464
693235	MIR92B	NR_030281.1	MIR92B	0.0028723933716857998
768239	PSAPL1	NM_001085382.1	PSAPL1	0.002904197951432086
118425	GDEP	NR_026555.1	GDEP	0.0029104742057124177
		Hs.162932	HS.162932	0.0031252062777078807
		Hs.559820	HS.559820	0.003222428963051623
100133034	LOC100133034	XR_036886.1	LOC100133034	0.0032667673919331147
		Hs.561960	HS.561960	0.003384888778334856
642412	LOC642412	XM_925931.1	LOC642412	0.0035227020471639255
100134821	LOC100134821	XM_001722581.1	LOC100134821	0.003624282268300971
340970	LOC340970	XR_038494.1	LOC340970	0.003737685755019992
		Hs.114286	HS.114286	0.003932674181302355
768222	MIR770	NR_030528.1	MIR770	0.003986096141096122
100131897	LOC100131897	XM_001725433.1	LOC100131897	0.004047894840157547
158825	LOC158825	XM_944306.1	LOC158825	0.004123734367948094
		Hs.541921	HS.541921	0.00413122853488739
729291	LOC729291	XR_041493.1	LOC729291	0.0041748180800655005
286310	LOC286310	XM_938575.1	LOC286310	0.004247249772839345
649021	LOC649021	XM_938105.1	LOC649021	0.0043309613155418465
6339	SCNN1D	NM_002978.2	SCNN1D	0.004384104476889884
9148	NEURL	NM_004210.3	NEURL	0.004404169604086631
100129272	LOC100129272	XR_038735.1	LOC100129272	0.004489042421780521
646686	LOC646686	XM_929633.1	LOC646686	0.0045888199572226215
100134245	LOC100134245	XM_001723619.1	LOC100134245	0.004651492454884095
729759	OR4F29	NM_001005221.2	OR4F29	0.004687841476130208
		Hs.579243	HS.579243	0.004778268630255688
400804	LOC400804	NR_024236.1	LOC400804	0.004843756848718989
	ERCC-00019	ERCC-00019	ERCC-00019	0.004868380227138507
		Hs.555115	HS.555115	0.0048744360081739
		Hs.545650	HS.545650	0.004878823751515827
		Hs.560651	HS.560651	0.004922635228871089
643195	LOC643195	XM_926563.1	LOC643195	0.004978023243253226
647323	LOC647323	XM_930391.1	LOC647323	0.005000769305957843

345	APOC3	NM_000040.1	APOC3	0.005101164742546303
642384	LOC642384	XM_926149.1	LOC642384	0.005173983519467021
56134	PCDHAC2	NM_031883.2	PCDHAC2	0.005303901459912222
100132515	LOC100132515	XM_001719495.1	LOC100132515	0.0053358302717896695
729623	LOC729623	XM_001716155.1	LOC729623	0.005393654110446185
127077	OR2T11	NM_001001964.1	OR2T11	0.005415934852160404
651007	LOC651007	XM_940119.1	LOC651007	0.005429676243793426
81931	ZNF93	NM_031218.2	ZNF93	0.005455575321176081
651481	LOC651481	XM_944705.1	LOC651481	0.0055417632881616195
100133612	LOC100133612	NR_024455.1	LOC100133612	0.005662336973006867
		Hs.129329	HS.129329	0.005768818496994594
84141	FAM176A	NM_001135032.1	FAM176A	0.005810056386037936
652264	LOC652264	XM_941675.1	LOC652264	0.005858098708048516
729459	LOC729459	XM_001720731.1	LOC729459	0.005911584917413475
		Hs.290834	HS.290834	0.005924131281531853
100132318	LOC100132318	XM_001718904.1	LOC100132318	0.0059987630165777885
389730	FAM75A6	XR_041507.1	FAM75A6	0.006002113715609757
		Hs.147725	HS.147725	0.006082745949588517
402160	LOC402160	XM_938047.1	LOC402160	0.00618058117796696
54550	NECAB2	NM_019065.2	NECAB2	0.006269455344883447
401898	ZNF833	NM_001013691.1	ZNF833	0.006362520312844321
10655	DMRT2	NM_181872.1	DMRT2	0.006384440067591239
139542	LOC139542	XM_066752.1	LOC139542	0.0064241876819909885
		Hs.505855	HS.505855	0.006434400433945601
650439	LOC650439	XM_944199.1	LOC650439	0.006508297260284593
1112	FOXN3	XM_929939.1	FOXN3	0.00660337190070171
407021	MIR29A	NR_029503.1	MIR29A	0.006642280164138524
		Hs.540289	HS.540289	0.006958649946345595
100134638	LOC100134638	XM_001719382.1	LOC100134638	0.0069592213315213935
339906	PRSS42	NM_182702.1	PRSS42	0.007160306544423903
121599	SPIC	NM_152323.1	SPIC	0.007388445760845789
649385	LOC649385	XM_938457.1	LOC649385	0.007497483289329452
23443	SLC35A3	NM_012243.1	SLC35A3	0.007553044335781477
442147	LOC442147	XM_498029.2	LOC442147	0.007648291765228071
100130701	LOC100130701	XM_001717180.1	LOC100130701	0.007827397078180115
653053	LOC653053	XM_925853.1	LOC653053	0.007900204294587978
29986	SLC39A2	NM_014579.1	SLC39A2	0.007999477533355752
8521	GCM1	NM_003643.2	GCM1	0.00812388029222538
122183	FLJ40296	XM_939203.1	FLJ40296	0.008183868937692471
		Hs.560728	HS.560728	0.008286770271927719
9837	GINS1	NM_021067.2	GINS1	0.008296728324984135
100131243	LOC100131243	XR_042510.1	LOC100131243	0.008446767342929243
693219	MIR634	NR_030364.1	MIR634	0.008456173083631108
100313772	MIR548M	NR_031667.1	MIR548M	0.00849958265815995
		Hs.551062	HS.551062	0.008645163460421596
		Hs.516420	HS.516420	0.00889648558077197

56834	GPR137	NM_020155.2	GPR137	0.00893880514031665З
199699	DAND5	NM_152654.2	DAND5	0.008942142967801789
84467	FBN3	NM_032447.3	FBN3	0.00898446467340792
652665	LOC652665	XM_001719814.1	LOC652665	0.009055477529238248
728654	LOC728654	Hs.578787	LOC728654	0.00905675591396397
654117	LOC654117	XM_939660.1	LOC654117	0.009082287421969534
148738	HFE2	NM_213653.2	HFE2	0.0091178306291845
6340	SCNN1G	NM_001039.2	SCNN1G	0.00911900427873428
5017	OVOL1	NM_004561.2	OVOL1	0.009159824466742588
		Hs.582355	HS.582355	0.009196764808675737
		Hs.581967	HS.581967	0.009287204096533039
649094	LOC649094	XM_945141.1	LOC649094	0.009407504706926069
5100	PCDH8	NM_002590.2	PCDH8	0.009772616370694803
645039	LOC645039	XM_928095.1	LOC645039	0.009787421976979783
2277	FIGF	NM_004469.2	FIGF	0.009833563822276537
9104	RGN	XM_937211.1	RGN	0.009849569716599147
113146	AHNAK2	NM_138420.2	AHNAK2	0.009922939294713589
120227	CYP2R1	NM_024514.4	CYP2R1	0.01001708226445753
652505	LOC652505	XM_941974.1	LOC652505	0.010040278019144642
		Hs.540000	HS.540000	0.010058940716288397
284618	C1orf104	NM_001039517.1	C1ORF104	0.010193965306880588
130612	TMEM198	NM_001005209.1	TMEM198	0.010292867538476691
		Hs.582338	HS.582338	0.010306764806367223
114784	CSMD2	NM_052896.2	CSMD2	0.010320462285929569
653623	LOC653623	XM_932876.1	LOC653623	0.010339962262382542
648993	LOC648993	XM_932049.1	LOC648993	0.010645850219767923
389400	GFRAL	NM_207410.1	GFRAL	0.01069802979537872
5651	PRSS7	NM_002772.1	PRSS7	0.01074816405737597
401551	WDR38	XM_941914.1	WDR38	0.010901481221334513
378948	RBMY1B	NM_001006121.1	RBMY1B	0.010973341854028786
27127	SMC1B	NM_148674.3	SMC1B	0.01098107373278998
100130808	LOC100130808	XM_001719331.1	LOC100130808	0.011022411175326036
8324	FZD7	NM_003507.1	FZD7	0.011119660122521448
100129055	LOC100129055	NR_024524.1	LOC100129055	0.011190951965361953
644311	LOC644311	XM_933386.1	LOC644311	0.011245518853591773
81469	OR2G3	NM_001001914.1	OR2G3	0.01125014975576129
	ERCC-00112	ERCC-00112	ERCC-00112	0.011616489420920842
642468	LOC642468	XM_931123.1	LOC642468	0.011628890636355619
642273	FAM110C	NM_001077710.1	FAM110C	0.01165792905924736
		Hs.520328	HS.520328	0.011873977183376822
		Hs.504674	HS.504674	0.011964393260100225
645126	LOC645126	XM_928158.1	LOC645126	0.011969802328208998
431704	RGS21	NM_001039152.2	RGS21	0.012066177424885915
653799	LOC653799	XM_930003.1	LOC653799	0.012084751428363714
		Hs.561963	HS.561963	0.01211700673958969
		Hs.519022	HS.519022	0.012308192274700338

2671	GFER	NM_005262.2	GFER	0.012358644008364584
27445	PCLO	NM_033026.5	PCLO	0.012435428150906784
374900	ZNF568	NM_198539.2	ZNF568	0.01251107186987248
51666	ASB4	NM_145872.1	ASB4	0.012561012785904813
728701	LOC728701	XM_001732848.1	LOC728701	0.01272238204011093
26257	NKX2-8	NM_014360.2	NKX2-8	0.012760908430736332
652148	LOC652148	XM_945224.1	LOC652148	0.012772870119896178
100130592	LOC100130592	XM_001725751.1	LOC100130592	0.012811240982860801
		Hs.539714	HS.539714	0.012840912412962124
57569	ARHGAP20	NM_020809.2	ARHGAP20	0.013118470760808389
653665	LOC653665	XM_928758.1	LOC653665	0.013128469298894354
4710	NDUFB4	NM_004547.4	NDUFB4	0.013222649280353434
401940	PRAMEF3	XM_001713666.1	PRAMEF3	0.013249479371121564
654466	KGFLP2	NR_003670.1	KGFLP2	0.013290672798568059
402217	LOC402217	XM_926343.1	LOC402217	0.013309286116595792
		Hs.529442	HS.529442	0.013367123475068342
84626	KIAA1862	NM_032534.1	KIAA1862	0.013422970670634833
		Hs.222909	HS.222909	0.013500050558039321
		Hs.278303	HS.278303	0.013528025823074474
79541	OR2A4	NM_030908.1	OR2A4	0.013535774011872056
23629	BRD7P3	NR_002730.2	BRD7P3	0.0136581493075215
642655	LOC642655	XM_926114.1	LOC642655	0.01368705768083874
222545	GPRC6A	NM_148963.1	GPRC6A	0.013711233775200095
401224	AACSL	XM_376454.3	AACSL	0.01371367905687376
6276	S100A5	NM_002962.1	S100A5	0.01379519022527498
90987	ZNF251	XM_937814.1	ZNF251	0.013899350375122767
285962	FLJ40852	NM_173677.1	FLJ40852	0.014060434770464255
728181	LOC728181	Hs.577605	LOC728181	0.0141347940546377
81543	LRRC3	NM_030891.3	LRRC3	0.014323459527928824
26254	OPTC	NM_014359.3	OPTC	0.014352304331036903
		Hs.553088	HS.553088	0.014374538074530693
406890	MIRLET7G	NR_029660.1	MIRLET7G	0.01440899484424025
646698	LOC646698	XM_929644.1	LOC646698	0.01483732667353354
147687	ZNF417	NM_152475.1	ZNF417	0.01493098172105505
574537	UGT2A2	NM_001105677.1	UGT2A2	0.01503029185309763
644452	LOC644452	XM_927592.1	LOC644452	0.015181770414054904
647527	LOC647527	XM_942881.1	LOC647527	0.015201527808483101
440337	LOC440337	NM_001013705.1	LOC440337	0.015425289593572807
768212	MIR758	NR_030406.1	MIR758	0.015508613507342983
727894	LOC727894	XM_001720317.1	LOC727894	0.015543022881295037
650433	LOC650433	XM_928063.1	LOC650433	0.015552972852532355
3375	IAPP	NM_000415.1	IAPP	0.015621056486094994
648684	LOC648684	XM_937754.1	LOC648684	0.01565313143237827
128239	IQGAP3	NM_178229.3	IQGAP3	0.015658724162056693
8120	AP3B2	NM_004644.3	AP3B2	0.0156638685342592
100127886	LOC100127886	XM_001721104.1	LOC100127886	0.01569002017637976

100129520	LOC100129520	XM_001720546.1	LOC100129520	0.015726804775336712
81797	OR12D3	NM_030959.2	OR12D3	0.015879234124184034
442204	LOC442204	XM_941158.1	LOC442204	0.015897567172426633
648879	LOC648879	XM_937958.1	LOC648879	0.01599569702802101
653392	LOC653392	XM_929544.1	LOC653392	0.016044495811424156
		Hs.454935	HS.454935	0.016147535401897353
		Hs.169896	HS.169896	0.016207540339586237
9469	CHST3	NM_004273.2	CHST3	0.016243901989474365
		Hs.552917	HS.552917	0.0162697160542665
653759	LOC653759	XM_933689.1	LOC653759	0.016371098321163312
645769	LOC645769	XM_928769.1	LOC645769	0.016510185576198752
7781	SLC30A3	NM_003459.4	SLC30A3	0.016511028193686883
80144	FRAS1	NM_206841.1	FRAS1	0.016523873196868023
664617	MIR542	NR_030399.1	MIR542	0.016613714909317213
100302134	MIR1289-2	NR_031621.1	MIR1289-2	0.01662531022456159
4134	MAP4	NM_002375.3	MAP4	0.016775584222864293
677775	SCARNA5	NR_003008.2	SCARNA5	0.016785208842328966
100133602	LOC100133602	XR_036856.1	LOC100133602	0.01683666670821988
400352	LOC400352	XR_016578.2	LOC400352	0.017079739864692106
653803	LOC653803	XM_376278.3	LOC653803	0.017127976599654482
645643	LOC645643	XM_933022.1	LOC645643	0.017150239866511565
441488	LOC441488	XM_929997.1	LOC441488	0.017215380981883834
644076	GLYCAM1	XM_945144.1	GLYCAM1	0.01722458438549089
646399	LOC646399	XM_929326.1	LOC646399	0.01754998407582004
652760	LOC652760	XM_942393.1	LOC652760	0.017582158167762218
100133056	LOC100133056	XM_001723741.1	LOC100133056	0.017614278666529242
653513	LOC653513	XM_927826.1	LOC653513	0.01764358092608569
100133638	LOC100133638	XM_001714231.1	LOC100133638	0.01773533627268364
29113	C6orf15	NM_014070.1	C6ORF15	0.017803645740275556
653149	LOC653149	XM_926213.1	LOC653149	0.01787715309412106
653941	LOC653941	XM_943151.1	LOC653941	0.018263229409780438
8385	OR1D4	NM_003552.2	OR1D4	0.018307855268222265
649280	LOC649280	XM_941486.1	LOC649280	0.018391792044690356
83856	FSD1L	NM_031919.1	FSD1L	0.01846573577669781
		Hs.579259	HS.579259	0.018467485733116545
		Hs.581657	HS.581657	0.018606211149496413
		Hs.133257	HS.133257	0.018767528532055853
100048912	ANRIL	Hs.556821	ANRIL	0.01879524479320668
692200	SNORD85	NR_003066.1	SNORD85	0.018976701195394474
		Hs.581645	HS.581645	0.01899843200332198
79710	MORC4	NM_024657.2	MORC4	0.01910590570428638
122038	LOC122038	XM_062912.1	LOC122038	0.019196424317865713
100128838	LOC100128838	XM_001724524.1	LOC100128838	0.01922384593465647
		Hs.242159	HS.242159	0.019287226676571085
729528	PRAMEF14	NM_001099854.1	PRAMEF14	0.019416555497664207
406902	MIR10A	NR_029608.1	MIR10A	0.019425208689276614

650977	LOC650977	XM_935578.1	LOC650977	0.01956314166256176
		Hs.541600	HS.541600	0.019629559223353542
2525	FUT3	NM_000149.1	FUT3	0.019700089768871422
729393	LOC729393	XM_935556.1	LOC729393	0.019865272887790345
100132209	LOC100132209	XM_001714087.1	LOC100132209	0.020221726568477035
652575	LOC652575	XM_942082.1	LOC652575	0.02028866877821377
100313884	MIR548H4	NR_031680.1	MIR548H4	0.020330775601036345
653806	LOC653806	XM_930050.1	LOC653806	0.020337837904343084
		Hs.563518	HS.563518	0.020396989331975476
10060	ABCC9	NM_020298.2	ABCC9	0.02040799559747524
646574	LOC646574	XM_933715.1	LOC646574	0.02062140321117068
730196	LOC730196	XM_930127.1	LOC730196	0.020624550982535607
650721	LOC650721	XM_944342.1	LOC650721	0.02062949543418754
642692	LOC642692	XM_926143.1	LOC642692	0.020863646281524408
		Hs.571515	HS.571515	0.020882015379294242
55908	LOC55908	NM_018687.3	LOC55908	0.02090164536976935
		Hs.534913	HS.534913	0.021066473132055688
643922	LOC643922	XM_927182.1	LOC643922	0.02111492999640619
441795	LOC441795	XM_937553.1	LOC441795	0.02113572373628669
		Hs.550218	HS.550218	0.02113799599016684
649041	LOC649041	XM_938124.1	LOC649041	0.021141231059488303
284417	TMEM150B	XM_209187.5	TMEM150B	0.02123943361465546
462	SERPINC1	NM_000488.2	SERPINC1	0.021275751939699005
441016	LOC441016	XM_936464.1	LOC441016	0.021327771230071232
56675	NRIP3	NM_020645.1	NRIP3	0.021359967827102422
		Hs.409403	HS.409403	0.021368339724236827
10512	SEMA3C	NM_006379.2	SEMA3C	0.021410594450011266
65082	VPS33A	NM_022916.3	VPS33A	0.021667463211417614
344558	SH3MD4	XM_293090.5	SH3MD4	0.02171773623940785
219557	MGC26647	NM_152706.2	MGC26647	0.02173921534576355
92105	INTS4	XM_941208.1	INTS4	0.02174200017972866
286557	RBMY1A3P	NR_001547.1	RBMY1A3P	0.02176637253887792
729240	PRR20C	NM_001130405.1	PRR20C	0.021780497537751933
158852	CT45-2	NM_152582.3	CT45-2	0.02184468944229154
646823	LOC646823	XM_933959.1	LOC646823	0.021954614523768918
51804	SIX4	NM_017420.2	SIX4	0.02196686864294639
100131696	LOC100131696	XM_001719335.1	LOC100131696	0.021973367354500152
		Hs.576474	HS.576474	0.021982121554966757
643937	LOC643937	XM_927195.1	LOC643937	0.02199470986446304
100131999	LOC100131999	XM_001714361.1	LOC100131999	0.02210884720074493
		Hs.543340	HS.543340	0.02221949537497687
728022	LOC728022	XM_001720082.1	LOC728022	0.022536695804648364
100134292	LOC100134292	XM_001717524.1	LOC100134292	0.022562678768112604
85302	FBF1	XM_946190.1	FBF1	0.02267592886449689
643062	LOC643062	XM_926433.1	LOC643062	0.02270477174650738
256144	OR4C3	NM_001004702.1	OR4C3	0.02274452747329593

388468	POTEC	NM_001137671.1	POTEC	0.022857498615990433
284467	FAM19A3	NM_001004440.1	FAM19A3	0.022993737812894953
5549	PRELP	NM_002725.3	PRELP	0.023129210919753312
643365	FLJ44054	NR_024609.1	FLJ44054	0.023168962721932574
51090	PLLP	NM_015993.1	PLLP	0.023229776752251536
55025	FLJ20712	XM_929740.1	FLJ20712	0.02332271643085656
55890	GPRC5C	NM_022036.2	GPRC5C	0.02333918703099564
5731	PTGER1	NM_000955.2	PTGER1	0.02334109658809124
100134687	LOC100134687	XM_001725648.1	LOC100134687	0.023364433029817847
6534	SLC6A7	NM_014228.2	SLC6A7	0.02351409087980999
153218	SPINK5L3	XM_376433.2	SPINK5L3	0.023561990750792117
389396	C6orf140	XM_001717222.1	C6ORF140	0.02380808919026948
148766	LOC148766	XM_944043.1	LOC148766	0.023814290697377855
161357	MDGA2	NM_182830.2	MDGA2	0.0239783457626738
100128074	LOC100128074	XM_001716711.1	LOC100128074	0.024034324339671117
389888	LOC389888	XM_372248.3	LOC389888	0.02420778796700878
3854	KRT6B	NM_005554.2	KRT6B	0.024242414229249387
253650	FLJ35740	NM_147195.1	FLJ35740	0.024248226617046308
9620	CELSR1	NM_014246.1	CELSR1	0.02442422597179391
650689	LOC650689	XM_944331.1	LOC650689	0.024620891735010586
650851	LOC650851	XM_939939.1	LOC650851	0.02470620850525972
		Hs.445179	HS.445179	0.024783874247643473
100131060	LOC100131060	XM_001726569.1	LOC100131060	0.024789556725427153
79837	PIP4K2C	NM_024779.3	PIP4K2C	0.024790272965445812
643431	LOC643431	XM_928128.1	LOC643431	0.0248750546433254
389365	LOC389365	XM_371797.2	LOC389365	0.02499211911817448
727884	LOC727884	XR_037459.1	LOC727884	0.02512510114659658
149708	WFDC5	NM_145652.2	WFDC5	0.025211307697428416
644054	FAM25C	NM_001137548.1	FAM25C	0.025286841884306046
206338	FLJ90650	NM_173800.3	FLJ90650	0.025331849080773603
345274	SLC10A6	NM_197965.1	SLC10A6	0.025539266030957946
574508	MIR505	NR_030230.1	MIR505	0.025542229322075727
677830	SNORA50	NR_002980.1	SNORA50	0.025648115404393816
		Hs.538367	HS.538367	0.0258033480835266
644316	LOC644316	XM_927484.1	LOC644316	0.0258203783413274
100128717	LOC100128717	XR_037607.1	LOC100128717	0.02594124537372537
93659	CGB5	NM_033043.1	CGB5	0.02604889505318224
		Hs.548213	HS.548213	0.026209602842411143
643402	LOC643402	XM_926737.1	LOC643402	0.02622565874116249
117531	TMC1	NM_138691.2	TMC1	0.026310984504829084
5303	PIN4	NM_006223.2	PIN4	0.026349367250758066
767610	SNORD114-29	NR_003222.1	SNORD114-29	0.02636118469746321
393076	LOC393076	XM_935699.1	LOC393076	0.02642569718895187
644098	LOC644098	XM_931998.1	LOC644098	0.026472465676084144
391819	KRT18P42	XR_039234.1	KRT18P42	0.02653184971257089
100129376	LOC100129376	XM_001714929.1	LOC100129376	0.026682345819321992

157724	SLC7A13	NM_138817.2	SLC7A13	0.026698560476787658
29931	LOH3CR2A	NM_013343.1	LOH3CR2A	0.026792621726054573
56122	PCDHB14	NM_018934.2	PCDHB14	0.026817705068609882
100131183	LOC100131183	XR_039203.1	LOC100131183	0.02688306877348543
		Hs.353831	HS.353831	0.026919042069835364
5080	PAX6	NM_000280.2	PAX6	0.027014121380323875
144402	CPNE8	NM_153634.2	CPNE8	0.027083771991721642
		Hs.580251	HS.580251	0.027115028419016078
728656	LOC728656	XM_932849.1	LOC728656	0.02719021068000494
652326	LOC652326	XM_941750.1	LOC652326	0.027209804441805464
648064	LOC648064	XM_937115.1	LOC648064	0.027538570723816908
		Hs.564082	HS.564082	0.02763550270793093
245973	ATP6V1C2	NM_144583.3	ATP6V1C2	0.027694136772679557
130120	REG3G	NM_001008387.1	REG3G	0.027932711689056387
340561	MGC42638	XM_926284.1	MGC42638	0.028277961439309544
3714	JAG2	NM_145159.1	JAG2	0.02841786844924962
		Hs.542618	HS.542618	0.028503835503501194
26492	OR8G2	NM_001007249.1	OR8G2	0.0285351290547875
		Hs.221951	HS.221951	0.028565419239745177
		Hs.538525	HS.538525	0.028632798570462893
574493	MIR520H	NR_030215.1	MIR520H	0.02865016994231157
9180	OSMR	NM_003999.1	OSMR	0.029033257307868314
		Hs.529590	HS.529590	0.029095528736048856
100129264	LOC100129264	XM_001725448.1	LOC100129264	0.029164465075395617
		Hs.232520	HS.232520	0.02932739677614784
79823	C2orf34	NM_024766.1	C2ORF34	0.02943581684894508
84530	SRRM4	NM_194286.2	SRRM4	0.029557850541321092
390880	LOC390880	XM_372707.3	LOC390880	0.029577098030507207
		Hs.544707	HS.544707	0.029829129270451688
348801	LNP1	XM_379203.2	LNP1	0.02985157069630891
		Hs.571245	HS.571245	0.029888463512216464
403257	OR4C45	NM_001005513.1	OR4C45	0.029893456666116217
100131573	LOC100131573	XM_001716335.1	LOC100131573	0.029921462243786185
642867	LOC642867	XM_931130.1	LOC642867	0.02995179201679189
574460	MIR498	NR_030182.1	MIR498	0.02999828744136964
5130	PCYT1A	NM_005017.2	PCYT1A	0.03001622031954481
6359	CCL15	NM_004167.3	CCL15	0.030086532136797735
693191	MIR606	NR_030337.1	MIR606	0.030092216058631844
130951	C2orf65	NM_138804.2	C2ORF65	0.030141646111764182
8542	APOL1	NM_145344.1	APOL1	0.03014373088034522
114038	C21orf84	NM_153752.1	C21ORF84	0.030270306908501375
		Hs.576698	HS.576698	0.030320096011271686
402066	LOC402066	XM_377725.3	LOC402066	0.030385264422877218
220032	GDPD4	NM_182833.1	GDPD4	0.030427690838538468
11245	GPR176	NM_007223.1	GPR176	0.030603100342009137
389442	LOC389442	XM_945400.1	LOC389442	0.030698112698738667

148811	PM20D1	NM_152491.3	PM20D1	0.030930813297524267
		Hs.568434	HS.568434	0.031003115105758768
391002	PRAMEF8	NM_001012276.1	PRAMEF8	0.031078148393666006
647954	LOC647954	XM_938141.1	LOC647954	0.03115141005922734
6003	RGS13	NM_144766.1	RGS13	0.03115524736341413
390616	ANKRD34C	XM_930512.1	ANKRD34C	0.03126601674428487
283585	LOC283585	XM_375099.2	LOC283585	0.03126958784080985
651787	LOC651787	XM_944941.1	LOC651787	0.03128112310360932
		Hs.46693	HS.46693	0.03129196190008781
		Hs.537983	HS.537983	0.03139569345078746
645848	LOC645848	XM_933202.1	LOC645848	0.03145673574418213
645201	LOC645201	XM_928236.1	LOC645201	0.031545488210135406
643446	LOC643446	XM_932755.1	LOC643446	0.0315889226603109
1014	CDH16	NM_004062.2	CDH16	0.0317473861882028
100129536	LOC100129536	XR_038075.1	LOC100129536	0.031820383098682216
441250	TYW1B	XR_015176.2	TYW1B	0.03183031116099876
644669	LOC644669	XM_927774.1	LOC644669	0.0318303814739753
		Hs.546079	HS.546079	0.03191419153433756
643799	LOC643799	XM_929213.1	LOC643799	0.031949486116225896
340895	C10orf112	XM_001716843.1	C10ORF112	0.032093992948235735
651923	LOC651923	XM_945017.1	LOC651923	0.0321414742003836
650632	LOC650632	XM_939721.1	LOC650632	0.03219295571905057
100302229	MIR1250	NR_031652.1	MIR1250	0.03222536563546908
648898	LOC648898	XM_944772.1	LOC648898	0.03224153084769567
677840	SNORA71D	NR_003018.2	SNORA71D	0.03228973382905253
		Hs.566864	HS.566864	0.032351696758506805
387755	INSC	NM_001031853.2	INSC	0.032364003360592905
127064	OR2T12	NM_001004692.1	OR2T12	0.032808211355858825
114771	PGLYRP3	NM_052891.1	PGLYRP3	0.032837605539625184
		Hs.581365	HS.581365	0.032875995067218604
11260	XPOT	NM_007235.3	XPOT	0.03293121754836928
440026	TMEM41B	NM_015012.1	TMEM41B	0.033056690688610224
400958	LOC400958	XM_379100.3	LOC400958	0.03307097076308331
100129000	LOC100129000	XM_001719583.1	LOC100129000	0.033091916558510376
652833	LOC652833	XM_942519.1	LOC652833	0.03321298535418646
149775	GNASAS	NR_002785.2	GNASAS	0.03344199943180014
653781	LOC653781	XM_929706.1	LOC653781	0.03347996462830669
222183	FLJ37078	NM_153043.3	FLJ37078	0.03354247547865309
100134372	LOC100134372	XM_001714877.1	LOC100134372	0.03355176427687743
643435	LOC643435	XM_931545.1	LOC643435	0.03357003327282396
5874	RAB27B	NM_004163.3	RAB27B	0.033575194307435996
79954	NOL10	NM_024894.1	NOL10	0.0335990779953308
84793	MGC12982	NR_026878.1	MGC12982	0.03361970373140142
286075	ZNF707	NM_173831.2	ZNF707	0.03371320701560756
100131320	LOC100131320	XM_001723688.1	LOC100131320	0.03385445543039937
283025	C10orf40	NR_024340.1	C10ORF40	0.03394751501046586

5599	MAPK8	NM_002750.2	MAPK8	0.03399866452797381
644079	LOC644079	XM_001720206.1	LOC644079	0.0341912882375524
368	ABCC6	NM_001079528.1	ABCC6	0.03431493026050804
100132767	LOC100132767	XM_001720251.1	LOC100132767	0.03440080915221909
138881	OR1L8	NM_001004454.1	OR1L8	0.03456597396989249
6584	SLC22A5	NM_003060.2	SLC22A5	0.03460459391804023
677829	SNORA49	NR_002979.2	SNORA49	0.03463335854931575
		Hs.574252	HS.574252	0.03477897443857523
728876	LOC728876	Hs.207162	LOC728876	0.03482274997843915
119467	CLRN3	NM_152311.1	CLRN3	0.035018728475164886
79822	ARHGAP28	NM_030672.2	ARHGAP28	0.03505231068410014
		Hs.562032	HS.562032	0.03508937672895057
7767	ZNF224	NM_013398.1	ZNF224	0.03512398245050982
5916	RARG	NM_000966.3	RARG	0.035124813290261965
100128537	LOC100128537	XM_001714326.1	LOC100128537	0.03517421150909691
		Hs.542875	HS.542875	0.03517734679208625
		Hs.18849	HS.18849	0.03527114019290617
100132972	LOC100132972	XR_039130.1	LOC100132972	0.035333362585726155
		Hs.549487	HS.549487	0.0354015803965153
93986	FOXP2	NM_148898.1	FOXP2	0.03550743036656206
284697	BTBD8	NM_183242.1	BTBD8	0.03557651653273978
81629	TSSK3	NM_052841.3	TSSK3	0.03559026459232305
728193	LOC728193	XR_001254.1	LOC728193	0.03560005450194582
55214	LEPREL1	NM_018192.2	LEPREL1	0.03561315135191362
100132963	LOC100132963	XM_001714657.1	LOC100132963	0.035694192240537256
	ERCC-00017	ERCC-00017	ERCC-00017	0.035822810531763706
10143	CLEC3A	NM_005752.2	CLEC3A	0.0358258784540761
100131673	LOC100131673	XM_001723856.1	LOC100131673	0.03586836841115992
692108	SNORD67	NR_003056.1	SNORD67	0.035873412006882904
643927	LOC643927	XM_927188.1	LOC643927	0.03590837992065841
729643	LOC729643	XM_001130893.1	LOC729643	0.03611177583217427
654101	LOC654101	XM_939354.1	LOC654101	0.03611818282844728
641311	RPL31P11	XR_000997.1	RPL31P11	0.03615679052967315
		Hs.545364	HS.545364	0.036224827502454135
646764	LOC646764	XM_929715.1	LOC646764	0.036249539928655795
727997	LOC727997	XM_001127849.1	LOC727997	0.03635395411914439
		Hs.542667	HS.542667	0.03639857127404468
		Hs.541092	HS.541092	0.03644836231965023
728924	LOC728924	XM_001132979.1	LOC728924	0.036626538623253165
652335	LOC652335	XM_941764.1	LOC652335	0.03689505668917005
26583	DUX2	NM_012147.2	DUX2	0.03698973758707169
344382	LOC344382	XM_293026.5	LOC344382	0.03700152462800427
100133053	LOC100133053	XM_001720766.1	LOC100133053	0.037011044365361706
642018	LOC642018	XM_936088.1	LOC642018	0.037069802753291296
643549	FLJ40606	XR_041973.1	FLJ40606	0.03716768958347956
401317	LOC401317	XM_938320.1	LOC401317	0.037207267570032204

100128485	LOC100128485	XM_001715311.1	LOC100128485	0.037552837763810396
		Hs.545648	HS.545648	0.03783155531545804
642194	LOC642194	XM_942778.1	LOC642194	0.03789201700212872
654032	LOC654032	XM_939494.1	LOC654032	0.03813728856527247
643414	LIPL2	XM_937207.1	LIPL2	0.03817870804042538
692086	SNORD17	NR_003045.1	SNORD17	0.038200153388503036
647080	LOC647080	XM_934321.1	LOC647080	0.03830985050841766
	ERCC-00053	ERCC-00053	ERCC-00053	0.03834869514975889
		Hs.534809	HS.534809	0.03834879151926415
57683	KIAA1571	XM_937234.1	KIAA1571	0.038358552981103154
729041	LOC729041	XM_001717661.1	LOC729041	0.03836846629500259
100127887	LOC100127887	XM_001723461.1	LOC100127887	0.03853325132203725
6048	RNF5	NM_006913.2	RNF5	0.03860009093862527
286464	CXorf59	NM_173695.1	CXORF59	0.038680235450205415
340549	LOC340549	XM_293332.1	LOC340549	0.038917475446315276
255411	LOC255411	XM_932014.1	LOC255411	0.03895963998255315
391365	SULT6B1	NM_001032377.1	SULT6B1	0.03911302437449217
651763	LOC651763	XM_940981.1	LOC651763	0.039244630984538384
389031	LOC389031	XM_940482.1	LOC389031	0.03926998584035779
338662	OR8D4	NM_001005197.1	OR8D4	0.0393256779732204
256281	NUDT14	NM_177533.2	NUDT14	0.03936138517869517
338751	OR52L1	NM_001005173.1	OR52L1	0.03952706246953922
55037	PTCD3	NM_017952.4	PTCD3	0.03965831209140964
730050	LOC730050	XM_942380.1	LOC730050	0.03970261977275519
649469	LOC649469	XM_938551.1	LOC649469	0.039726795675571205
647651	LOC647651	XM_936697.1	LOC647651	0.03976741733921677
100133418	LOC100133418	XM_001714459.1	LOC100133418	0.039798961546514564
		Hs.143909	HS.143909	0.03987953435966447
		Hs.541752	HS.541752	0.04004451312984737
147945	NLRP4	NM_134444.3	NLRP4	0.04005249679157248
6288	SAA1	NM_000331.2	SAA1	0.040065832203235015
		Hs.547175	HS.547175	0.040153296946881016
8074	FGF23	NM_020638.2	FGF23	0.04016990512673844
441381	LRRC24	NM_001024678.1	LRRC24	0.04029216676542084
728343	LOC728343	XM_939774.1	LOC728343	0.040324070434557116
		Hs.475950	HS.475950	0.0403536263781617
375010	LOC375010	XM_927556.1	LOC375010	0.040404601611003954
4656	MYOG	NM_002479.3	MYOG	0.04050658101039191
		Hs.437179	HS.437179	0.04052765359085568
116449	CLNK	NM_052964.2	CLNK	0.0405367112071025
3060	HCRT	NM_001524.1	HCRT	0.04065663865807545
26740	OR1J2	NM_054107.1	OR1J2	0.04079535607325396
146177	LOC146177	XM_370944.4	LOC146177	0.04082276324038739
400965	LOC400965	NM_001013677.1	LOC400965	0.040887347474745844
		Hs.580126	HS.580126	0.0410471418128887
		Hs.545887	HS.545887	0.04111865623111575

727827	LOC727827	XM_001127359.2	LOC727827	0.04112645889694715
135656	DPCR1	NM_080870.2	DPCR1	0.04113050080090478
647515	LOC647515	XM_001718676.1	LOC647515	0.04116426887393105
100133348	LOC100133348	XM_001716396.1	LOC100133348	0.04126059807045334
1447	CSN2	NM_001891.1	CSN2	0.04130424464274184
647197	LOC647197	XM_934499.1	LOC647197	0.04144851834200355
		Hs.157621	HS.157621	0.041452897550665085
285479	LOC285479	XM_211908.5	LOC285479	0.04162774662655042
23430	TPSD1	NM_012217.2	TPSD1	0.04162903602256395
100130123	LOC100130123	XM_001726844.1	LOC100130123	0.04178163825145163
		Hs.545230	HS.545230	0.0418944246284238
641978	LOC641978	XM_935752.1	LOC641978	0.04193526858697818
		Hs.583274	HS.583274	0.0419771000983454
284099	C17orf78	NM_173625.3	C17ORF78	0.0420299255226024
284451	ODF3L2	NM_182577.1	ODF3L2	0.042116097623423256
		Hs.569313	HS.569313	0.04213676251389568
148645	MGC40168	NM_153709.1	MGC40168	0.042325609228867814
50945	TBX22	NM_016954.2	TBX22	0.042337420790947905
		Hs.560736	HS.560736	0.042560307781693854
100129343	LOC100129343	XM_001724611.1	LOC100129343	0.0426296711237734
100129514	LOC100129514	XM_001724518.1	LOC100129514	0.042757097797527295
645218	LOC645218	XM_945368.1	LOC645218	0.042789979520314755
83481	EPPK1	XM_926068.1	EPPK1	0.0428175486064293
3547	IGSF1	NM_001555.2	IGSF1	0.04284432572336439
258010	SVIP	NM_148893.1	SVIP	0.042926970724047234
646280	LOC646280	XM_929223.1	LOC646280	0.043143966073033654
51802	ACCN5	NM_017419.1	ACCN5	0.043346582974570357
		Hs.538535	HS.538535	0.04341198215012938
		Hs.562701	HS.562701	0.04362372092389725
441501	FLJ46446	XM_001715413.1	FLJ46446	0.04370004734540954
54985	HCFC1R1	NM_001002017.1	HCFC1R1	0.0437394289594447
653060	LOC653060	XM_925779.1	LOC653060	0.043789209896504436
		Hs.135587	HS.135587	0.04379133238179812
		Hs.40289	HS.40289	0.04400621524649907
652762	LOC652762	XM_945940.1	LOC652762	0.044008620289967326
100132966	LOC100132966	XM_001714155.1	LOC100132966	0.04407882716170898
692057	SNORD12	NR_003030.1	SNORD12	0.044299546532056915
26812	SNORD37	NR_002602.1	SNORD37	0.04433768044660433
728116	LOC728116	NM_144621.2	LOC728116	0.04442996576954535
55049	C19orf60	NM_001100419.1	C19ORF60	0.04453832178515606
100128356	LOC100128356	XM_001719204.1	LOC100128356	0.04462339465347257
100124542	SNORA11E	NR_003712.1	SNORA11E	0.04473602678130252
100129119	LOC100129119	XM_001718523.1	LOC100129119	0.04499965944222805
81282	OR51G2	NM_001005238.1	OR51G2	0.04508569441179958
		Hs.582113	HS.582113	0.0451131786661592
		Hs.537150	HS.537150	0.04516681361170144

51555	PEX5L	NM_016559.1	PEX5L	0.04524256321740555
100130370	LOC100130370	XM_001715409.1	LOC100130370	0.045377009541867616
		Hs.442504	HS.442504	0.04540920889661434
442425	FOXB2	NM_001013735.1	FOXB2	0.04542397134263472
644152	LOC644152	XM_927360.1	LOC644152	0.04542523508126548
		Hs.563147	HS.563147	0.04546159300769052
		Hs.537608	HS.537608	0.04562692161699765
653434	LOC653434	XM_933966.1	LOC653434	0.04568770829349333
100124540	SNORA11C	NR_003710.1	SNORA11C	0.04576946351814093
		Hs.555208	HS.555208	0.045770937282462607
26245	OR2M4	NM_017504.1	OR2M4	0.04578649557143999
401109	FLJ25996	NM_001001699.1	FLJ25996	0.04589285003980043
100130141	LOC100130141	XR_038164.1	LOC100130141	0.046055871031270804
126868	C1orf161	NM_152367.1	C1ORF161	0.04609199594523736
285512	FAM13AOS	NR_002806.2	FAM13AOS	0.046145373406548514
402317	OR2A42	NM_001001802.1	OR2A42	0.04616272697550872
		Hs.541191	HS.541191	0.04625642254718605
374739	TEPP	NM_199046.1	TEPP	0.04625830589891997
285697	LOC285697	XM_941117.1	LOC285697	0.04632282358450559
		Hs.566890	HS.566890	0.04635531669736749
5155	PDGFB	NM_033016.1	PDGFB	0.04650986869654247
727836	LOC727836	XR_038572.1	LOC727836	0.04652114694423314
389730	LOC389730	XR_000527.1	LOC389730	0.046547519642591115
374378	GALNTL4	NM_198516.1	GALNTL4	0.046735817907034405
643037	LOC643037	XM_936787.1	LOC643037	0.04679494780096214
100302130	MIR1302-4	NR_031633.1	MIR1302-4	0.04686041799156694
649186	LOC649186	XM_938252.1	LOC649186	0.04686938946073637
		Hs.561430	HS.561430	0.046901961073004754
		Hs.302412	HS.302412	0.04694635264572553
8330	HIST1H2AK	NM_003510.2	HIST1H2AK	0.046960800231884994
		Hs.544495	HS.544495	0.04696506376407463
		Hs.574587	HS.574587	0.0469684078568494
100131244	LOC100131244	XM_001724055.1	LOC100131244	0.046981434770345706
		Hs.546135	HS.546135	0.046996587342102505
651177	LOC651177	XM_940301.1	LOC651177	0.04700075868907026
340602	CXorf67	NM_203407.1	CXORF67	0.047001076847879825
646570	LOC646570	XM_933711.1	LOC646570	0.04703739710584047
		Hs.550042	HS.550042	0.047115038681197076
1297	COL9A1	NM_001851.3	COL9A1	0.04714323250208604
10014	HDAC5	NM_001015053.1	HDAC5	0.04722777454918724
641995	LOC641995	XM_935775.1	LOC641995	0.047228437613621325
729374	LOC729374	XR_038569.1	LOC729374	0.04724643599108605
653786	LOC653786	NR_003676.2	LOC653786	0.04725527473065633
26780	SNORA68	NR_000012.1	SNORA68	0.047285746510851566
400019	LOC400019	XM_378349.3	LOC400019	0.047393136010094226
140691	TRIM69	NM_080745.3	TRIM69	0.0474119722132346

647234	LOC647234	XM_934553.1	LOC647234	0.047439423733171025
56163	RNF17	NM_031994.1	RNF17	0.04767407512859314
89932	PAPLN	NM_173462.2	PAPLN	0.04768950584794584
403314	APOBEC4	NM_203454.1	APOBEC4	0.04793759494161416
4842	NOS1	NM_000620.1	NOS1	0.048026600103485875
100126319	MIR216B	NR_030623.1	MIR216B	0.048043630203721495
		Hs.58089	HS.58089	0.048076218570047326
646358	LOC646358	XM_929287.1	LOC646358	0.048107642962684015
439941	C8orf54	XR_000579.1	C8ORF54	0.04814616528597775
1826	DSCAM	NM_206887.1	DSCAM	0.04833964991785896
651711	LOC651711	XM_940923.1	LOC651711	0.0483643092356878
		Hs.582594	HS.582594	0.0484084727751594
84809	CROCCL1	XR_039823.1	CROCCL1	0.048508335050976716
36	ACADSB	NM_001609.2	ACADSB	0.04852330528721858
149465	WDR65	NM_152498.1	WDR65	0.04853470869428911
652510	LOC652510	XM_941981.1	LOC652510	0.04863040057616678
1800	DPEP1	NM_004413.1	DPEP1	0.048648356765277206
5279	PIGC	NM_153747.1	PIGC	0.04869033866821056
645651	LOC645651	XM_928665.1	LOC645651	0.048761572597092004
644415	LOC644415	XR_018194.2	LOC644415	0.04887709279665332
154288	ECAT1	NM_001017361.1	ECAT1	0.04888972575962395
646187	LOC646187	XR_016991.2	LOC646187	0.04903448172268005
645196	LOC645196	XM_928233.1	LOC645196	0.04918479829182512
81607	PVRL4	NM_030916.1	PVRL4	0.0493173128212757
285193	DUSP28	NM_001033575.1	DUSP28	0.0493731738586513

References

1. Arber, D.A.; Orazi, A.; Hasserjian, R.; Thiele, J.; Borowitz, M.J.; Le Beau, M.M.; Bloomfield, C.D.; Cazzola, M.; Vardiman, J.W. The 2016 revision to the World Health Organization classification of myeloid neoplasms and acute eukemia. *Blood* **2016**, *127*, 2391–405.
2. Nepstad, I.; Hatfield, K.J.; Aasebø, E.; Hernandez-Valladares, M.; Brenner, A.K.; Bartaula-Brevik, S.; Berven, F.; Selheim, F.; Skavland, J.; Gjertsen, B.T.; et al. Two acute myeloid leukemia patient subsets are identified based on the constitutive PI3K-Akt-mTOR signaling of their leukemic cells; a functional, proteomic, and transcriptomic comparison. *Expert Opin. Ther. Targets* **2018**, *22*, 639–653.
